# High costs of capital shape the mitigation effects of renewable energy deployment globally

**DOI:** 10.1038/s41467-026-75666-6

**Published:** 2026-08-13

**Authors:** Luke Hatton, Gbemi Oluleye, Malte Jansen, Iain Staffell, Adam Hawkes

**Affiliations:** 1 Department of Chemical Engineering, Imperial College London, London, UK; 2 Columbia Centre on Sustainable Investment, Columbia University, New York, NY USA; 3 Centre for Environmental Policy, Imperial College London, London, UK; 4 Science Policy Research Unit, University of Sussex, Brighton, UK

**Keywords:** Developing world, Social sciences, Solar energy

## Abstract

High costs of capital are a major barrier to renewables deployment in low- and middle-income countries (LMICs), impeding decarbonisation efforts despite strong renewable resources. Using grid carbon intensity trajectories, we show that renewable deployment in LMICs has mitigation potentials over 20 times larger per project than high-income countries, even before accounting for planned fossil projects. High costs of capital increase renewable levelised costs of electricity (LCOEs) in LMICs substantially, adding an average of US$26/MWh (solar) and US$24/MWh (onshore wind) relative to high-income financing terms. Financing costs account for up to 78% of the LCOE in LMICs, compared with 37-39% in high-income countries, restricting a large portion of global renewable potential. Under a scenario consistent with the target of tripling global renewable capacity by 2030, this corresponds to additional annual costs of US$245bn (solar) and US$329bn (wind). Our results highlight the importance of reducing financing costs in LMICs for a more equitable and efficient energy transition.

## Abstract (web summary)

This study finds that low- and middle-income countries hold over 20 times the renewable mitigation potential of high-income countries, but higher financing costs increase renewable electricity costs by about US$25/MWh on average, highlighting the importance of affordable capital for an equitable energy transition.

## Introduction

Annual clean power investment flows need to rise globally to at least US$1.5 trillion per year between 2025 and 2030^1^ if the world is to achieve the UNFCCC goal agreed at COP28 of tripling global renewable capacity by 2030 to at least 11,000 GW^2^ and the Paris-aligned goal of net zero emissions by mid-century^3^. Whilst annual investment flows into clean power projects globally have risen rapidly over recent decades, they only reached US$780bn in 2025^4^. Existing flows are highly concentrated geographically, with China, Europe and North America accounting for nearly 80% of global annual investment into renewables^5^, but only covering 59% of global electricity demand^6^. In low- and middle-income countries (LMICs) outside of China, total energy investment has not risen in real terms since 2015^4^ and there is still a strong pipeline of planned fossil power projects in many regions^7^ (despite high-quality renewable resources).

Wind and solar photovoltaic (referred to hereafter as solar) are expected to contribute the majority of the 2030 tripling renewables target in terms of both generation and capacity^8^, and both have experienced notable cost reductions over recent decades that have exceeded most expert forecasts^9,10^. Cost reductions to date have had four major drivers: falling capital costs due to experience and economies of scale^11^; decreasing costs of capital from financial learning^12^ and periods of low interest rates^13^; technical innovations increasing the average capacity factor^14^; and lower operating and maintenance costs^15^. The extent to which these have led to reductions in regional levelised costs and thus deployment has varied^16^, with investment in LMICs also being constrained by skill shortages^17^, limited grid integration^18^, and inadequate policy and regulatory setups^19^. However, the principal barrier is insufficient flows of energy investment at affordable costs of capital^20–22^. From an investor’s perspective, there is a distinct lack of “bankable” projects, where the risk-return profile fits the investor’s risk appetite^23^. For commercial actors, larger returns are required for investments with higher perceived risks^24^, with the notable risk premiums demanded for projects in LMICs^25^ leading to the cost of capital for renewables being over twice as high in LMICs as in high-income countries^26,27^. High risk premiums for renewable projects in LMICs are driven by a range of factors at the project, sector and country levels^28^, with many risks being macroeconomic in nature^28^, including currency risks^29^, political instability^30^ and the strength (or perceived weakness) of state institutions^31^. These risk factors also drive the high premiums that LMICs pay for both public and private borrowing in sectors beyond clean energy, which cause difficulties in attracting external investment at affordable rates across sectors and are one of the drivers of the widening “financing gap”^32^.

Existing work has already highlighted the challenge that high costs of capital pose to the energy transition in LMICs^21,22,33^, leading many to become stuck in a “climate investment trap”^33^. Impacts on renewable deployment are compounded by the important role that finance plays in determining the cost of renewable electricity^34^, with the economic viability of renewables highly sensitive to financing costs^35–37^. Detailed exploration of this challenge is also hampered by poor data availability, with terms of finance regarded as privileged information in a competitive environment^38^. Cost of capital estimation methods with sectoral, national and temporal granularity are a key area of interest for energy economists and modellers^39–43^, as conclusions drawn from technoeconomic and energy system models have been shown to be highly sensitive to cost of capital assumptions^34,36,44^. Developing estimates of the likely ranges of costs of capital in LMICs and the extent to which they constrain technical resource potential is therefore important for ensuring the accuracy and utility of technical insights from modelling studies^34,42^, improving the relevance of modelled decarbonisation pathways^45^ and geospatial technoeconomic assessments^46^.

Marked differences in the geographic distribution of high-quality renewable energy resources and accessible costs of capital for project developers are a key driver of the geographic concentration of global deployment, often out of line with locations that have the best renewable potential^46^. Whilst this holds implications for the cost and equity of the global energy transition^21^, the implications on the overall effectiveness of mitigation efforts and progress towards the UNFCCC target of tripling renewables by 2030 have not yet been explored in depth. As LMICs often have higher current grid intensities and/or larger planned project pipelines of fossil power generation capacity, deploying renewables would deliver much larger mitigation per unit of deployed capacity than in advanced economies. Bringing down high costs of capital in LMICs and accelerating renewables deployment through de-risking efforts^47^ would be likely to lead to a more efficient decarbonisation of the global energy system both in terms of energy generation^48^ (by deploying capacity in locations with high capacity factors) and mitigation potential^49^ (by deploying capacity in locations with high and/or rising grid intensities). However, there has been limited examination of the interplay between renewable LCOEs, costs of capital and the lifetime mitigation potential of deployed renewables in the literature to date.

Here, using a global geospatial renewables model, we develop estimates of the levelised cost of electricity (LCOE), annual technical potential and lifetime carbon mitigation of solar, onshore wind and offshore wind power at a high geospatial granularity, using country- and technology-specific estimated costs of capital for 176 countries in 2026. Combining the technical and economic analysis with grid carbon intensity trajectories, we find that deploying wind and solar in LMICs could deliver over twenty times the lifetime mitigation per renewable project than in high-income countries, but that high costs of capital substantially increase renewable LCOE in LMICs, by an average of US$26 (solar) and US$24 (onshore wind) per MWh, slowing more effective progress towards achieving the UNFCCC renewables target of tripling renewable capacity through harnessing high-quality renewable potential in LMICs.

## Results

### Modelling the cost and scale of renewable potential

**Fig. 1:**
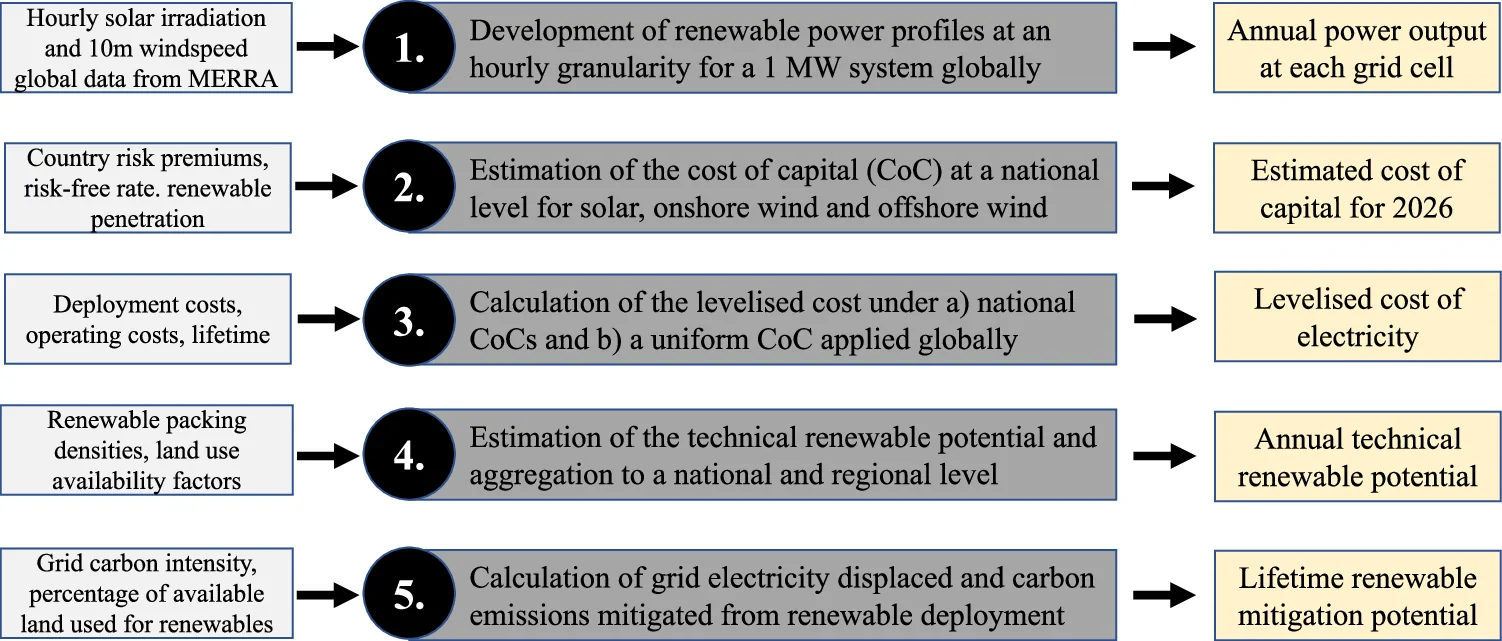
Schematic of the modelling approach used in this work. The five modelling steps in this work are outlined with corresponding inputs and outputs from each step. Inputs are highlighted in light grey on the left whilst the main outputs from each step are detailed in tan on the right. The approach is applied to solar, onshore wind and offshore wind separately at a global scale.

Our modelling approach entails five distinct steps, illustrated in Fig. 1. First, we develop hourly power output profiles of solar PV^50^ and wind power^51^ globally at a high geospatial granularity to accurately depict sub-national distributions of renewable potential. Secondly, we create estimates of country- and technology-specific costs of capital for 176 countries for 2026, adapting an existing, peer-reviewed approach to widen its scope and geographical coverage (see Methods)^39^. Thirdly, we calculate the LCOE based on national or regional deployment costs for solar and wind power, using national, technology-specific estimates of the cost of capital and evaluating the share of the LCOE attributable to financing costs at each grid cell. We also perform the calculations using a uniform cost of capital globally, based on the average estimated cost of capital in the UNFCCC Annex II countries for the relevant technology (referred to hereafter as high-income countries, though we note this is an outdated country categorisation). These two cost of capital assumptions used as a basis for estimation globally (referred to as the National and Uniform bases) were selected to illustrate the extent to which high costs of capital in LMICs push up renewable LCOEs, reducing the economic viability of renewables and so contributing to deployment being concentrated in high-income countries (where there are likely to be lower grid carbon intensities). Fourth we calculate the technical renewable potential (i.e., the maximum electricity production per grid cell per year) using land use availability factors and packing density limits for each renewable technology, which are then aggregated to both national and regional levels. Finally, the renewable mitigation potential at each location is also calculated using the technical potential, project lifetime and grid carbon intensity scenarios, which are used to generate LCOE-mitigation potential curves for each region under the National and Uniform cost of capital assumptions.

### Global renewable energy technical potentials

**Fig. 2:**
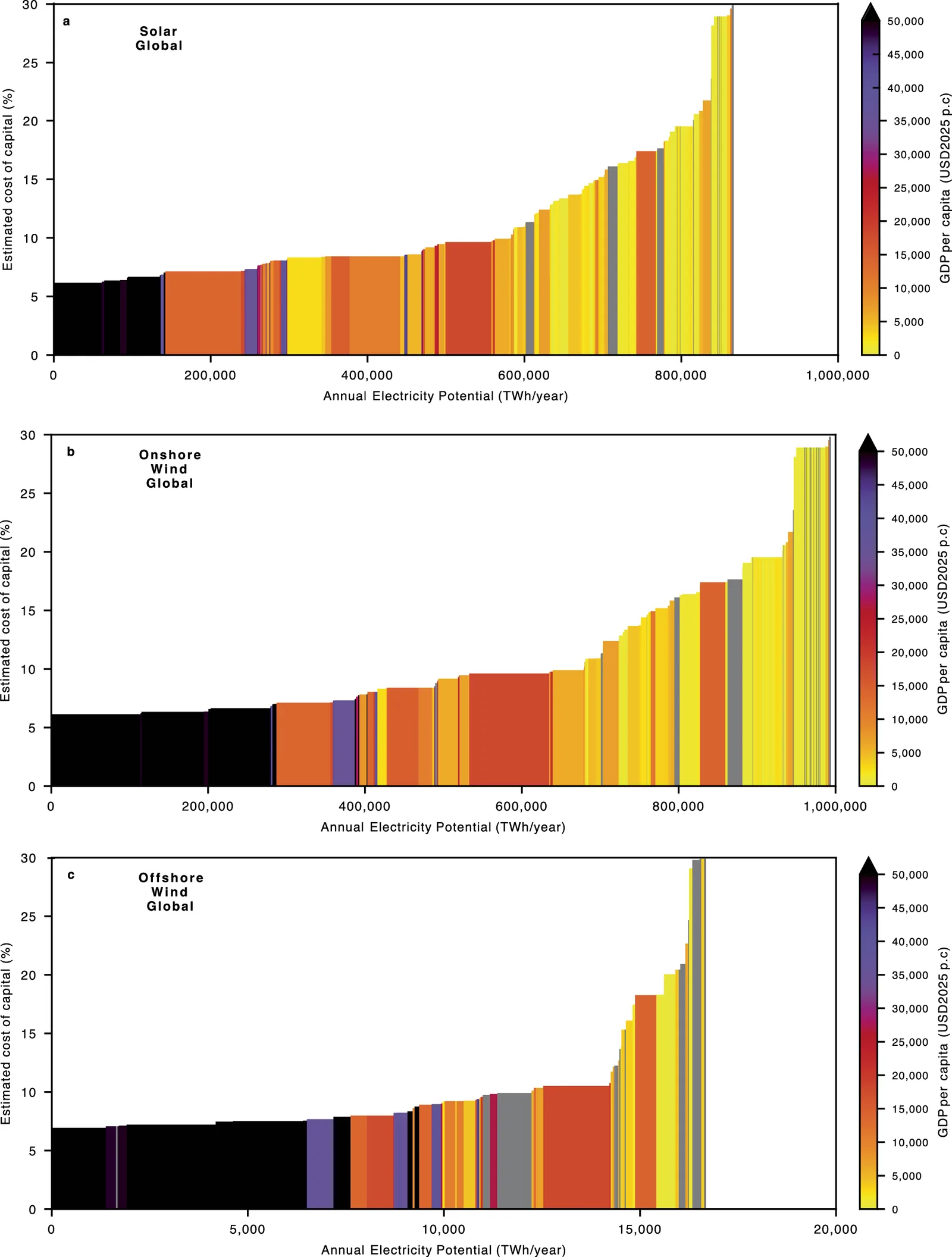
Global renewable technical potential curves, ordered by the estimated weighted average cost of capital for 2026. Results are plotted for **a** solar PV, **b** onshore wind and **c** offshore wind. Shading is used to indicate the level of economic development in each country, measured using GDP per capita in absolute terms and with potential aggregated up to a country level. Grey reflects countries with missing economic data. Packing density and land use constraints were applied to constrain technical potential at each location (see Methods). Minimum cut-offs of 10% and 18% average capacity factor were applied to the solar and wind potentials to reflect constraints in economic feasibility^53^. Note the different x-axis scale used for offshore wind potential compared with solar and onshore wind.

Figure 2 presents the annual solar, onshore wind and offshore wind technical potential globally, obtained by applying the model with minimum capacity factor cut-offs (see Methods) across over 96,000 locations. Using a geospatial LCOE model with such a high spatial granularity allows us to explore more effectively the effects of a) intra-country variations in renewable resource (i.e., capacity factor), b) land availability constraints that reduce the technical potential, and c) lifetime mitigation potential at each location at a higher granularity. Technical potentials are ordered here by the estimated national costs of capital, with GDP per capita in each country illustrated (see Supplementary Fig. 3 for regional breakdowns). Global estimates of the annual technical potentials are 860,000, 996,000 and 16,500 TWh per annum for solar, onshore and offshore wind, respectively, broadly in line with other estimates from the literature (e.g., the comparison of global solar and wind potential estimates by Chu et al.^52^).

Despite substantial technical potential (in many cases higher quality than high-income countries on a capacity factor basis), the majority of LMICs face costs of capital for renewables much higher than high-income countries, in line with findings from other work^28,39,43^. Estimated cost of capital range substantially across countries, from 6.1–30.6% (solar and onshore wind) and 7.0–31.6% (offshore wind) in nominal terms (see **Methods**). Commercial financing terms for renewable energy in LMICs are around two to three times the averages in high-income countries, which are 7.1% (solar and onshore wind) and 8% for offshore wind (see Supplementary Fig. 2 for the geographic distribution of costs of capital). Low-income countries (LICs) largely face the highest costs of capital, with a clear relationship between cost of capital and GDP per capita from the results presented in Fig. 2. High costs of capital necessitate support from international partners and financial institutions for the energy transition in LMICs, particularly for LICs, with an estimated USD 110 – 120 bn per annum in concessional financing required to achieve the UNFCCC renewables target of tripling capacity by 2030^53^.

### High costs of capital hold back cost-competitive renewable potential

**Fig. 3:**
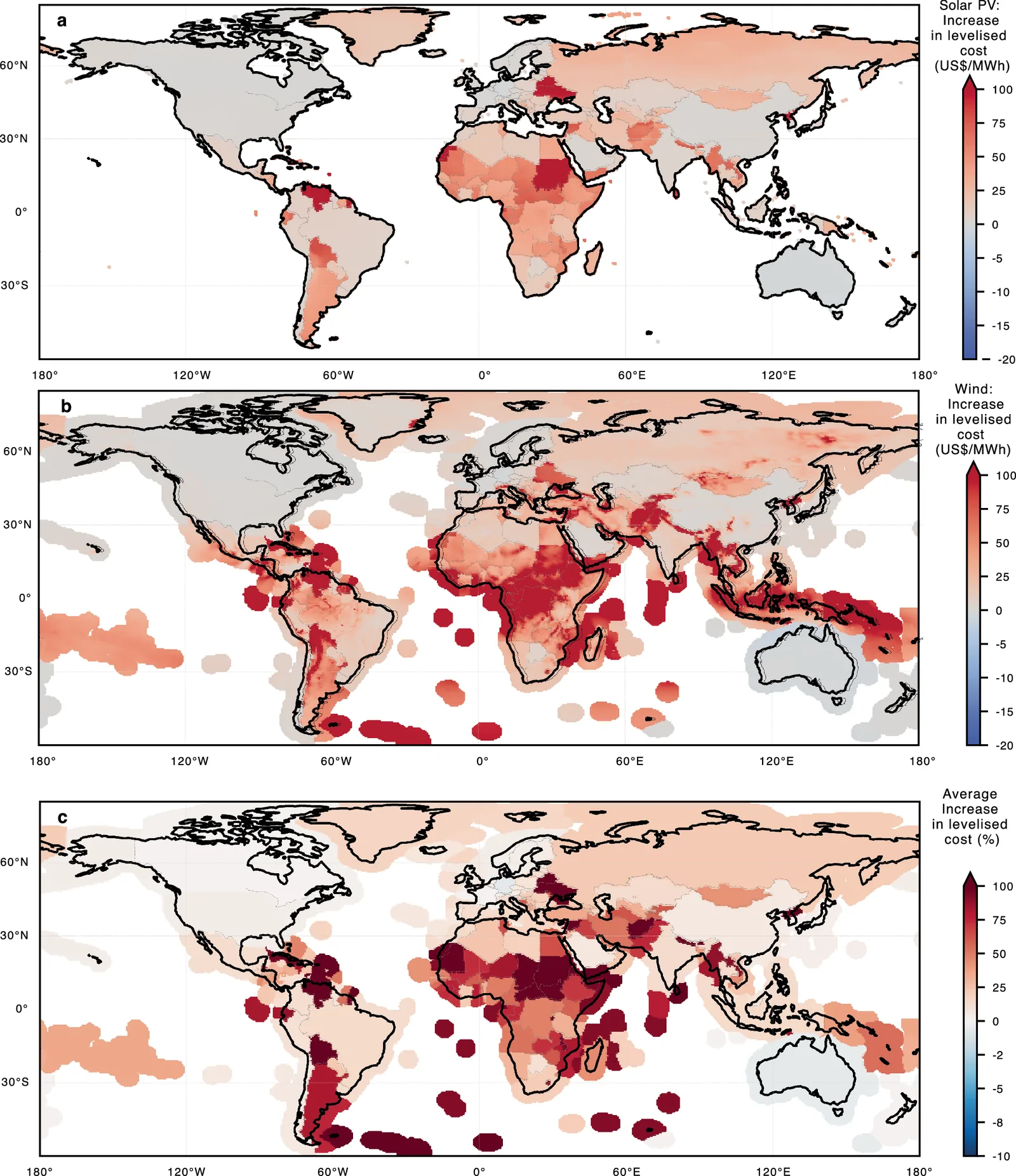
Changes in the levelised cost of electricity (LCOE) between the National and Uniform cost of capital assumptions. Results are plotted for **a** solar PV, **b** wind power and **c** the average change for both technologies, all in percentage terms. The increase in offshore wind is plotted, where appropriate, in b) and c). National uses country- and technology-specific estimates of the cost of capital for 2026, whilst Uniform uses a uniform cost of capital of 7.1% (solar and onshore wind) and 8% (offshore wind), respectively. These values are the average estimated costs of capital for 2026 in countries categorised by the UNFCCC as Annex II countries.

The levelised cost of electricity (LCOE) is one measure used to assess the competitiveness of electricity generation across different projects and technologies, and measures the unit price of electricity required for the projects to economically break even^16,54^. Using an economic model with regionally (and nationally, where available) varying deployment costs, the LCOE for solar, offshore wind and onshore wind was evaluated with a high spatial granularity using two cost of capital assumptions: 1) National, using technology- and country-specific cost of capital for 2026; and 2) Uniform, where the average cost of capital for 2026 in nominal terms in high-income countries for the relevant technology is applied uniformly across the globe. Figure 3 demonstrates the change in the LCOE from each technology between the National and Uniform calculations (spatial plots of LCOE under both assumptions are included in Supplementary Fig. 4 and Supplementary Fig. 5. Differences in the changes in the LCOE within countries are influenced by the distribution of renewable potential, with sites with poor renewable potential and high costs of capital seeing a larger increase in the LCOE (as the same change in cost of capital increases the LCOE in locations with lower capacity factors more). The average increase globally in LCOE under National costs of capital are US$26 and US$24/MWh for solar PV and onshore wind, respectively, an increase of 37% and 38%. For offshore wind, which involves larger capital expenditures, this rises to US$42/MWh (39%).  For all technologies, this could be enough to impair competitiveness against fossil power generation.

Delivering on the UNFCCC target of tripling global renewable capacity by 2030 requires an estimated additional 8,000 GW of renewable capacity to be deployed. In energy terms, this translates to around 23,000 TWh of renewable generation annually^3^ - mainly from onshore wind and solar PV - which is 2.3% (solar) and 2.7% (onshore wind) of the global technical potentials calculated in Fig. 2. Around 5,000 GW of the additional capacity needs to be concentrated in LMICs, based on modelling conducted by the IEA^55^. High costs of capital in LMICs could lead to an annual increase of up to US$329bn and US$248bn in costs for onshore wind and solar PV (US$6.6 and US$5.0 trillion across their lifetime), if the additional 5,000 GW were deployed in the best locations in LMICs (see Methods). Increases in LCOE under national costs of capital are highest in countries in Africa, Central Asia and parts of South America, exceeding US$150/MWh for wind and US$100/MWh for solar, whilst in Western Europe and North America there is little or no change in the LCOE (given that costs of capital are close to or equal to the averages in Annex II countries). Assessment of the breakdown of the cost of capital for renewables demonstrates that the increases in LMICs are driven almost exclusively by the country risk premiums, which measure perceived macroeconomic and political investment risks and are evaluated based on the sovereign credit rating produced by credit rating agencies (e.g., S&P, Moody’s, Fitch) for each country^31^. Strengthening institutions, addressing corruption and pursuing increased political stability - although not directly targeting infrastructure investments - can play a key role in improving the investment landscape by reducing the country risk premium and so the cost of capital for clean infrastructure investments.

**Fig. 4:**
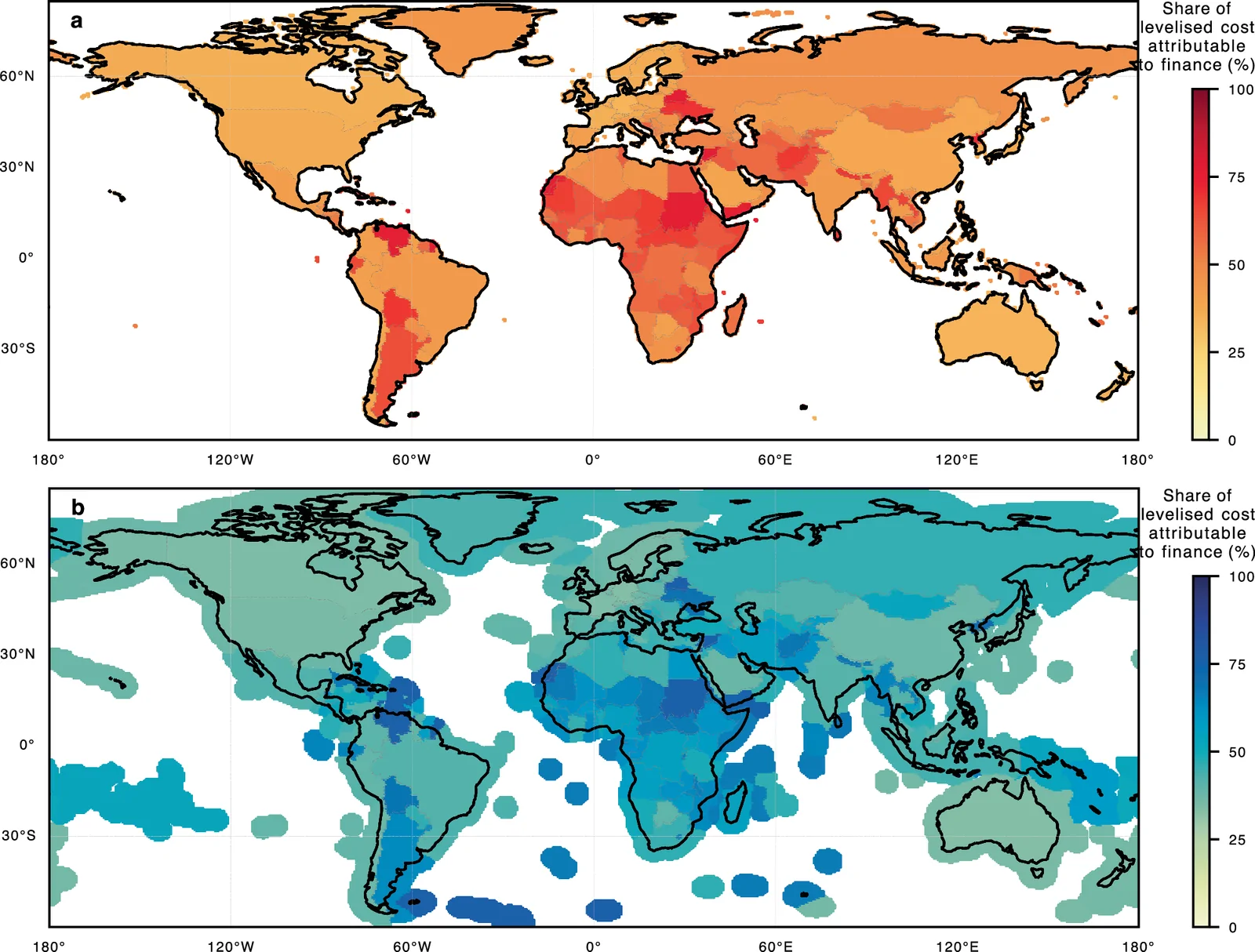
The share of the levelised cost of electricity (LCOE) attributable to the cost of capital. Shares are mapped globally for **a** solar PV and **b** wind power. Results are depicted for the National calculation, which uses country- and technology-specific costs of capital for 2026 to calculate the LCOE at each location. The share of financing costs is calculated by subtracting the contributions of capital costs and operating costs to the overall LCOE under a discount rate of zero, with the remaining share set as the proportion associated with financing costs.

Financing costs already make up a substantial portion of the overall LCOE for solar and wind in high-income countries^56^, due to their high capital intensities and low operational costs^36^. Figure 4 plots the share of the LCOE attributable to financing costs at each grid cell modelled, which ranges from 33–78% across technologies and regions under the National assumption. Under the Uniform assumption, in contrast, financing costs contribute an average of 33-36% to the LCOE across all technologies. In line with the geographic distribution of cost of capital, the lowest shares of finance in the LCOE under the National assumption are in locations in LMICs, with high costs of capital substantially pushing up the LCOE by increasing the share and scale of financing costs included in the LCOE^36^. Both results highlight that the LCOE is extremely sensitive to the cost of capital, as demonstrated in other studies^35–37^.

**Fig. 5:**
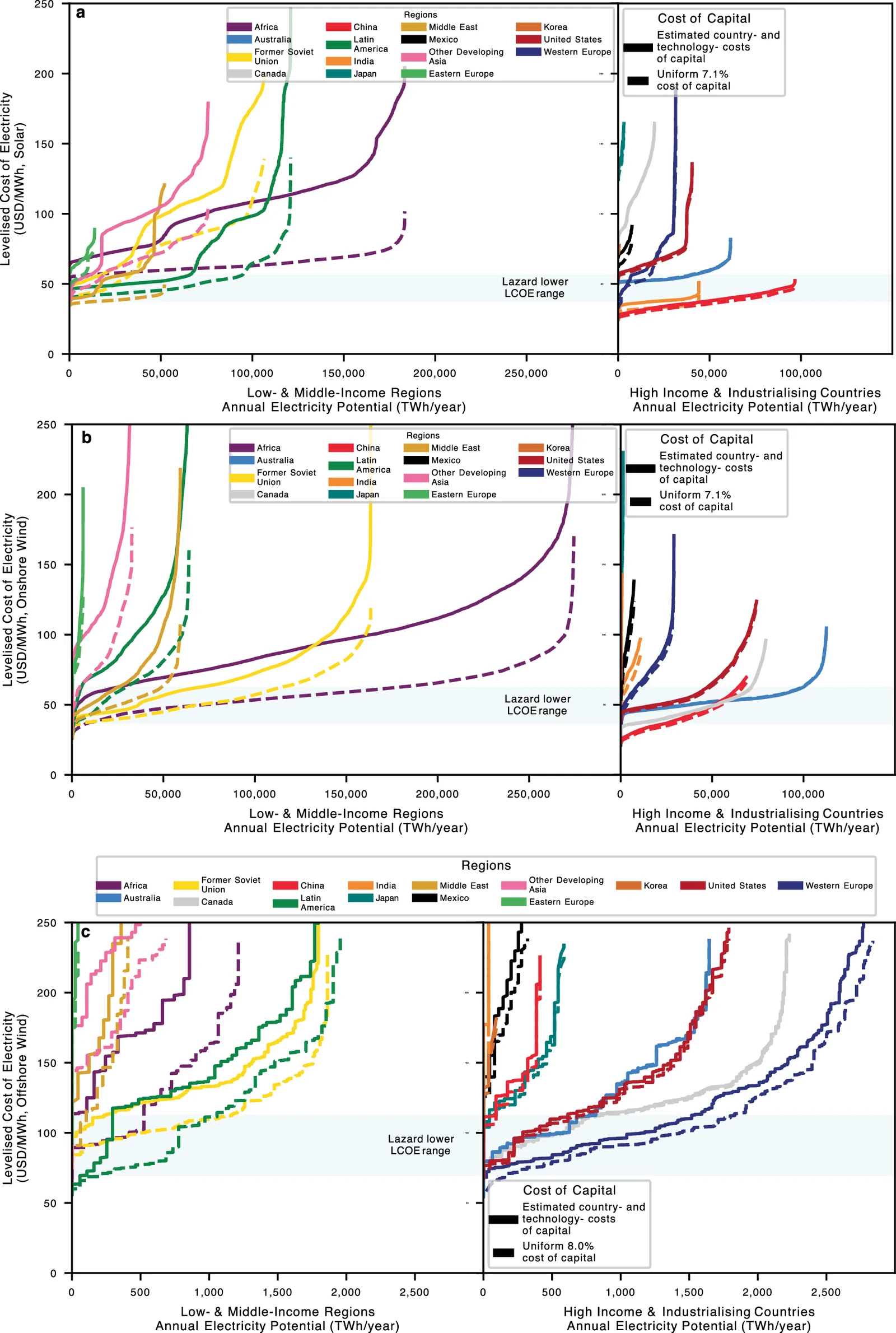
Regional levelised cost-supply curves, plotted for all 16 regions examined under the National and Uniform cost of capital assumptions. Curves are plotted for **a** solar PV, **b** onshore wind and **c** offshore wind. Note the different scale for electricity potential used for the offshore wind plot. Levelised costs under the national estimates of technology costs of capital are displayed using the solid lines, whilst the dotted lines depict the levelised cost supply curve in each region under the average technology cost of capital in the UNFCCC Annex II countries. The full country-region mappings are included in the Supplementary Material.

Understanding the extent to which low-cost renewable power can be scaled up, both nationally and regionally, is also important to long-term energy planning and to the effective design of policy and regulatory support to achieve the UNFCCC goal of tripling renewables by 2030^57^. Figure 5 plots regional LCOE-supply curves under both the National and Uniform assumptions for each technology, with regions grouped using the mappings from the TIAM-Global model^58^. The largest increases in LCOEs are in Africa, Latin America and the Former Soviet Union (FSU) regions, reaching up to US$150 and over US$150/MWh for solar and onshore wind, respectively (for locations within the minimum capacity factor bounds). The lowest achievable LCOEs globally are in China, reaching US$27 and US$19/MWh for solar and onshore wind in the best locations, due to a combination of low costs of capital and the lowest national deployment costs on a per-kW basis globally for both technologies^16^. For offshore wind, LCOEs reach as low as US$60/MWh in Western Europe, in line with offshore auction bids^59^.

Africa has the largest renewable potential for both solar PV and onshore wind, reaching over 183,000 and 274,000 TWh per annum (6.1 and 9.1 times the volume of electricity generated globally from all sources in 2023^3^). Low-cost renewable electricity generation in Africa is severely limited by high costs of capital, with high borrowing costs and subsequent debt servicing levels impairing investment and development on the continent more broadly^60^. The percentage of Africa's solar potential within or below Lazard’s solar LCOE benchmarks^61^ (US$38-78/MWh) falls from 97.1% to 15.5% under the National basis. For onshore wind, the share of technical potential within or below Lazard’s ranges (US$37-86/MWh) falls from 93.7% to 40%. Other LMIC regions also see similarly drastic falls in the share of their technical potential below the midpoints of the Lazard LCOE benchmarks under their costs of capital, particularly solar in the Middle East (100% to 53.5%), solar in Other Asia (74.1% to 23.2%) and solar (94.9% to 63.4%) and onshore wind (83.0% to 41.5%) in Central and South America.

Historically, the majority of offshore wind development has taken place in China and European countries bordering the North Sea^62^, reaching a global installed capacity of 72 GW in 2023^3^ (259 TWh per annum, using an average capacity factor of 41%^16^). Global offshore potential estimated in this work is nearly 60 times this value, reaching 16,500 TWh. However, the majority of locations evaluated here have offshore wind LCOEs far higher than the Lazard benchmarks^61^ (US$70–157/MWh), and IRENA’s reported global weighted average (US$75/MWh)^16^. The largest potential below the midpoint of Lazard’s benchmark (US$112/MWh^61^) is in Western Europe, at 1517 TWh per annum. If LMICs were able to access the same costs of capital as high-income countries, substantial offshore wind technical potentials could be accessed at costs below the benchmark, with 1058 and 1132 TWh per annum respectively in Central & South America and the Former Soviet Union regions.

### Renewable mitigation potential

**Fig. 6:**
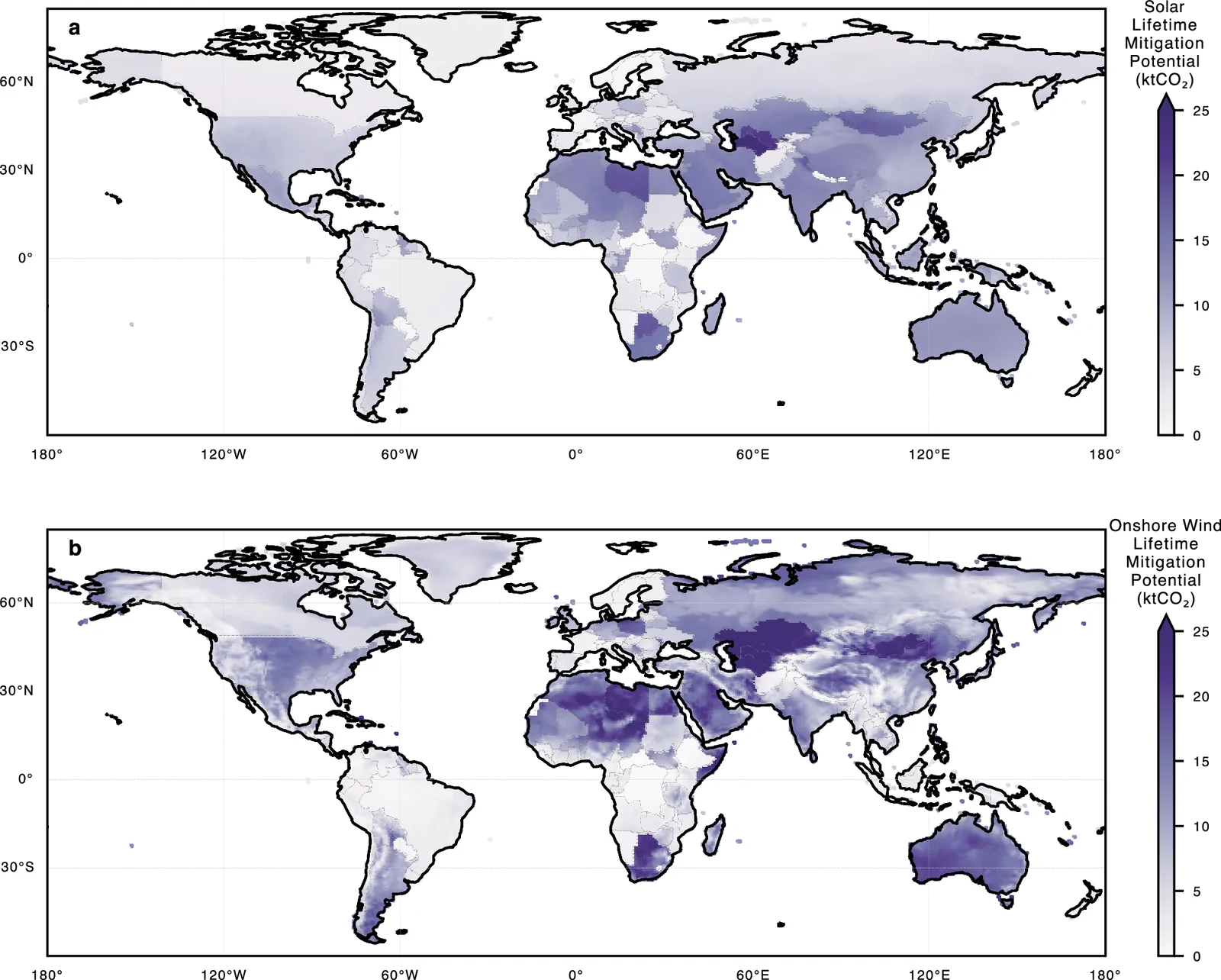
Lifetime carbon mitigation potential from the deployment of 1 MW of installed capacity. Results are plotted globally for **a** solar PV and **b** onshore wind, in kilotonnes of CO_2_. Calculations assume that each kWh of renewable electricity generated displaces a kWh of grid electricity and each project has a lifetime of 20 years. Grid carbon intensity is taken from Ember Climate^64^ for 2025 (with missing country data filled from 2021-2024 using the most recent year of data) and is assumed to fall linearly to zero carbon emissions (see Methods), based on the need for global power sector emissions to reach zero by 2045 to align with the Paris Agreement’s targets^3^. We note that country-specific contexts mean that grid carbon intensity is unlikely to fall linearly and in some countries (especially LMICs) it may increase in the short term, so the estimates presented here are likely to be conservative.

High costs of capital have implications for both the equity and pace of the energy transition^21^, as well as the effectiveness of global mitigation efforts. Meeting the UNFCCC target of tripling global renewable capacity by 2030 by deploying wind and solar in locations with high grid carbon intensities and higher capacity factors can deliver far larger renewable generation and emissions reductions per MW of installed capacity^49^. As it is macroeconomic and market-level factors that drive the cost of capital and investment flows for renewables, there are typically weak links between local renewable potential, national grid intensities and the cost of capital for renewables. Figure 6 plots the lifetime mitigation potential for 1 MW of wind or solar capacity deployment globally, based on current grid carbon intensities and Paris-aligned future grid intensity trajectories^63^ (see Methods). Countries with some of the highest mitigation potentials for both solar and wind include South Africa, Libya, Turkmenistan and Uzbekistan – all LMICs – at over 20 times the lifetime mitigation potential in high-income countries such as France, Norway, and the UK which have already decarbonised large portions of their electricity generation mix.

**Fig. 7:**
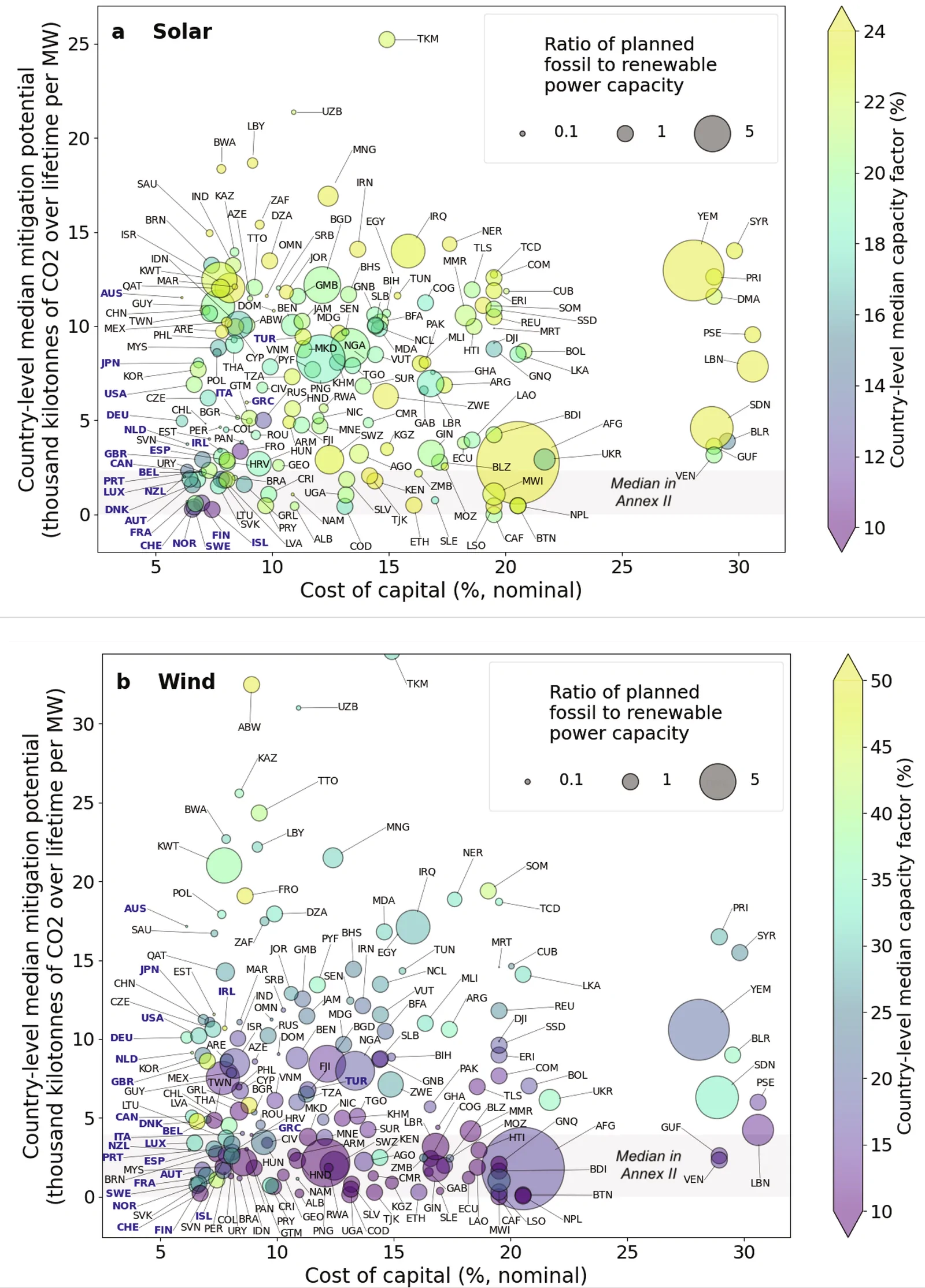
Country-level comparisons of the cost of capital, mitigation potential and planned fossil and renewable generation capacity additions. Results are mapped for **a** solar PV and **b** onshore wind. Each country is shown as a bubble on the scatter plot, with the median value presented for subnational parameters. The median capacity factor for each country is indicated through shading, whilst the size corresponds to the ratio of planned fossil power projects in MW to planned renewable capacity. Data for the planned project pipeline are taken from Global Energy Monitor 2026^66^. Countries are labelled using their ISO-3 country codes. Countries categorised as Annex II by the UNFCCC are labelled in bold font with navy blue colouring.

Excluding Australia, which still has substantial coal power on its grid^64^, it is predominantly LMICs that have the highest grid intensities (see **Supplementary)** and so are likely to have the highest lifetime mitigation effects from deployment of renewable capacity. We illustrate in Fig. 7 that it is largely LMICs where the project pipeline of fossil power exceeds renewables by mapping the cost of capital, mitigation potential and ratio of planned fossil to renewable power by country, demonstrating that solar and wind deployment in many LMICs can deliver much higher mitigation potentials per MW than the median in Annex II countries. Accounting for the future pipeline of planned fossil power additions^7^ – largely focused in LMICs – makes supporting renewable deployment in LMICs even more effective in mitigation terms. Many LMICs with strong renewable potential (measured through median capacity factor) that face high costs of capital have a much larger pipeline of fossil power generation projects than renewable projects, indicating that high costs of capital could be a factor holding back the deployment of renewables to meet energy demand growth. Without interventions to reduce the cost of capital, the realisation of the planned fossil project pipeline in LMICs could lead to fossil infrastructure lock-in, risking missing global emissions targets under the Paris Agreement. In some LMIC regions, limited renewable deployment despite ample potential has already led to growing fossil power capacity deployment. For example, despite having some of the strongest solar resources globally, the continent of Africa has seen a 52% increase in installed gas power capacity over the last decade^65^, with nearly 60 GW of gas power currently in development to meet future demand increases from economic and population growth^66^. Solar and wind could provide alternative power generation sources to meet rising electricity demand, but as we show here, their competitiveness is held back under high costs of capital in LMICs.

**Fig. 8:**
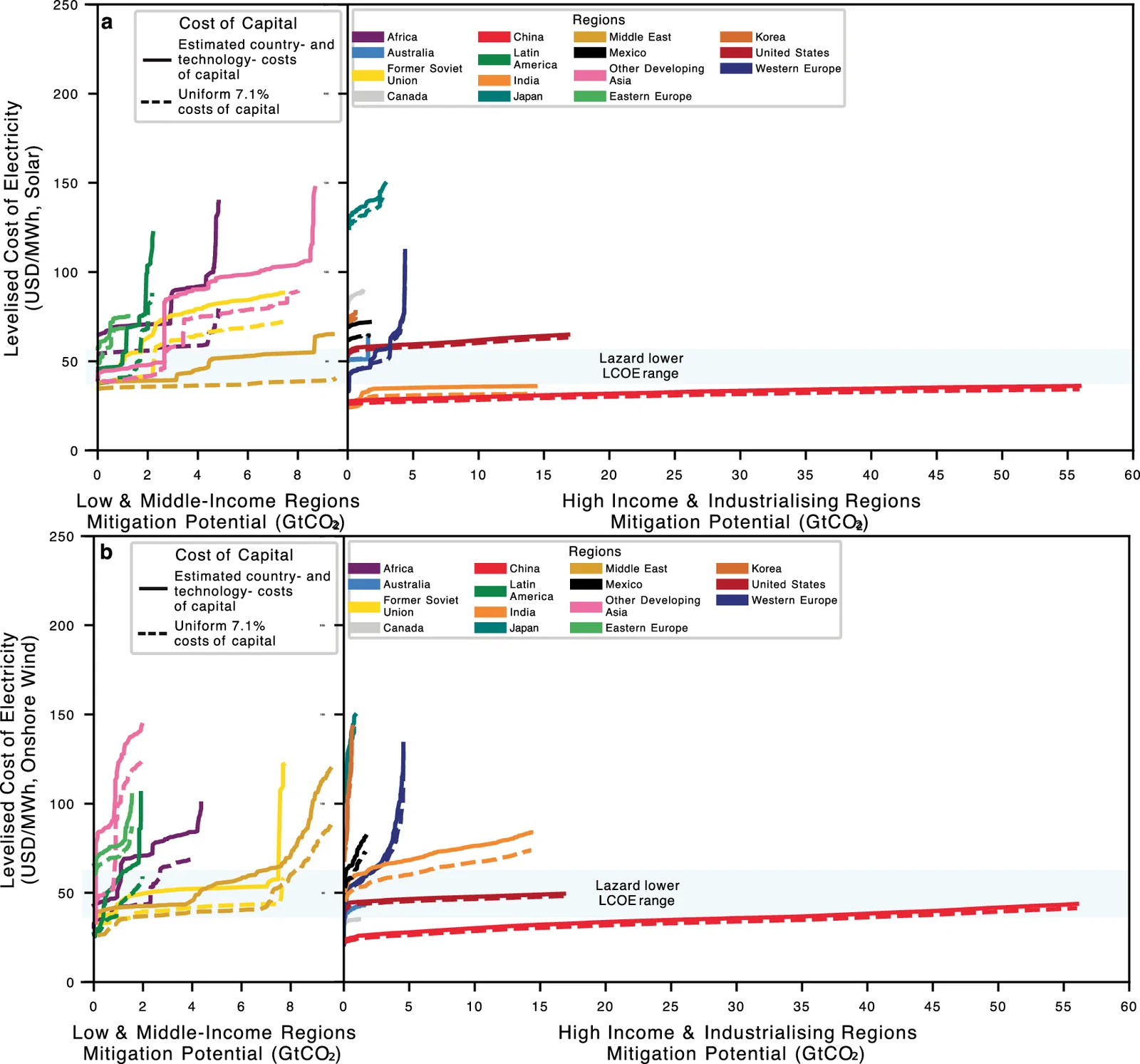
Lifetime technical mitigation potential, plotted at a regional level. Curves are shown for **a** solar PV and **b** onshore wind, in gigatonnes of carbon dioxide against the levelised cost of electricity under the National and Uniform assumptions. As with Fig. 6, calculations assume that each kWh of renewable electricity generated displaces a kWh of grid electricity, with the total deployment limited by the current electricity generation in each country. Grid carbon intensity and total electricity generation are taken from Ember Climate^63^. Cost-supply curves were restricted to USD 150/MWh to avoid a small number of locations with poor renewable resources and extremely high LCOEs distorting the end of the cost-supply curve.

LMICs need to play a larger role in achieving the COP28 tripling renewables target^55,67^, though installed capacity nationally may need to more than triple to sufficiently displace fossil power from national grids whilst meeting rapidly rising electricity demand. High costs of capital mean that the levels of mitigation required in LMICs largely cannot be met at competitive LCOEs, for both solar and onshore wind. Figure 8 plots the cumulative power sector mitigation potential against the estimated LCOE for solar and onshore wind at a regional level, with limits applied based on national annual electricity generation capacity in 2025 (see Methods). The largest mitigation potentials in the power sector are in India, the U.S. and China, rising up to 14, 16 and 55 GtCO_2_, with LCOEs largely within the Lazard LCOE benchmarks^61^ used as a reference point for US power generation costs. For LMICs, only in the Middle East and Latin America can a substantial portion of emissions be abated with solar at costs below the average benchmark of US$56/MWh, whilst for onshore wind, it is only a negligible portion of mitigation potential in Other Asia that is below the benchmark under National costs of capital. Access to lower costs of capital in high-income countries for Other Asia, Africa (solar) and Former Soviet Union (wind), brings a larger portion of mitigation potential within the benchmarks. However, even under the Uniform costs of capital for some regions a large share of power system mitigation potential remains above these benchmarks, highlighting the need for further cost reductions and support for emerging technologies to accelerate power system decarbonisation.

## Discussion

High costs of capital for renewables are a substantial barrier to energy transitions in LMICs, which could reduce the overall mitigation effects of meeting the target of tripling global renewable capacity by 2030 by impeding deployment in locations with high grid carbon intensities. Addressing high costs of capital through policy reform, de-risking efforts and the scaling of concessional finance is therefore essential to an equitable, rapid and effective global energy transition^28^. Here, through a technoeconomic evaluation of the technical potential, levelised cost and mitigation effects of solar, onshore and offshore wind generation globally, we show that high costs of capital are holding back the harnessing of high-quality renewable potential in low- and middle-income countries (LMICs). High costs of capital lead to an average increase in LCOEs of US$26, 24 and 42/MWh (for solar PV, onshore and offshore wind, respectively), compared to if LMICs could access financing on high-income country terms. Given that LMICs typically have much higher grid carbon intensities and pipelines of planned fossil projects, we show that the lifetime mitigation potential from deploying wind or solar power could be twenty times the levels in high-income countries, but that high LCOEs in those locations are a barrier to deployment.

Our modelling demonstrates that the cost of capital for renewable energy investments is also closely related to national GDP per capita, aligning with existing findings^68^. Low-income countries (LICs) therefore face the highest costs of capital, more than triple the average in high-income countries, increasing the share of financing costs in renewable LCOEs in LICs to up to 78%. High costs of capital lead to inadequate levels of clean energy investment in these countries^33^, putting renewables at a competitive disadvantage and leaving the economies least able to fund their energy transition continuing to depend on fossil fuels and retain vulnerabilities to price volatility^69^. For example, in Bangladesh, 62% of electricity demand growth has been met by expansion of gas power in the ten years to 2021. Rising LNG costs forced Bangladesh to halt purchases of LNG and implement load-shedding measures^70^. Expanding electricity supply through solar in Bangladesh could have saved US$2.7bn and reduced imports by 25%^71^, but our modelling shows that it faces a high cost of capital (13%), nearly double the average in high-income countries (7%). Failure to bring down the cost of capital in LMICs means that countries such as Bangladesh will be unable to access the economic benefits of falling renewable deployment costs^72^ and so will continue to be exposed to price shocks from geopolitical disruptions (e.g., those caused by the war in Iran in early 2026).

Addressing the barriers to sufficient flows of energy investment into LMICs at affordable costs of capital is not just motivated by a global energy justice perspective, though it is essential that this remains at the heart of the global response to climate change^73^. To avoid further lock-in of carbon-intensive infrastructure^69^ – which could lead to a failure to meet Paris Agreement-compliant global emissions trajectories and a subsequent risk of future asset stranding^74^ – ensuring that renewables are cost-competitive in LMICs is essential. LMICs are expected to account for a high share of the future growth in energy demand over the next few decades^28^. Lowering the cost of capital to ensure that demand growth is met predominantly by clean energy sources such as renewable will be key to avoiding fossil power lock-in, as well as ensuring that the additional 8000 GW of renewable capacity needed for the COP28 goal delivers both the maximum amount of clean electricity and tonnes of greenhouse gases emissions mitigated by displacing fossil power.

Estimates show that annual clean energy investment into LMICs will need to increase by more than six times by 2030 for the world to be on track for net zero by 2050^28^. Targeted efforts to bring down the cost of capital in LMICs are essential to scaling private investment flows, which our modelling suggests are largely held back by high country risk premiums. These conclusions align with the IEA's assessment that around 70% of the risk factors contributing to high costs of capital in LMICs are caused by macroeconomic and currency risks^28^, accounted for here in the country risk premiums. Factors influencing the country risk premium also include corruption, political structures, exposure to violence and the strength of legal systems, challenges that pose difficulties in attracting investments beyond  the energy sector. Developing strong enabling environments for investment is important to scaling private flows to levels required to meet the UN Sustainable Development Goals, including through strengthening domestic institutions, reforming legal systems and designing regulatory environments to promote transparency and fair competition. Existing programmes to support domestic policymakers in addressing these issues include the UNDP’s Governance for Anti-Corruption for Peaceful and Inclusive Societies Programme and the World Bank’s Improving Business Environment for Prosperity Programme. For investors, political risk insurance (e.g., through the World Bank’s MIGA programme) can also provide coverage against country-level risk factors, including expropriation and breaches of contract, whilst for project developers, a dedicated investment facilitation unit for renewable project permitting could substantially reduce their  exposure to corruption by streamlining approval pathways.

Country risk premiums are also largely derived from the host country’s sovereign credit rating, as investors and businesses typically do not have the ability to conduct their own detailed macroeconomic and political assessments across countries of interest. Sovereign credit ratings act as a hard “sovereign ceiling” where projects or corporations are typically unable to be rated higher than the country rating^75^, meaning that they are all but guaranteed a higher cost of capital than the sovereign borrowing costs. Any biases, inaccuracies or misperceptions of sovereign risk can therefore severely constrain project financing conditions under the “sovereign ceiling” effect, with existing work having demonstrated the subjectivity in rating decisions and identified systematic ratings biases below the ratings implied based on sovereign credit rating methodologies^76,77^. This bias has been partly attributed to geographic and institutional distance between rated LMICs and the major credit rating agencies, all of which are headquartered in Europe or North America. These issues point to potential structural biases in their methodology against LMICs^78^, underlining the need for reform of the structure, methodologies and role of credit rating agencies to help scale clean energy investments into LMICs^78,79^.

Default rates for private lending in LMICs are also substantially lower than the default rate implied by historical sovereign credit ratings^80^, particularly for infrastructure projects, demonstrating a potential need to decouple the link between project ratings and sovereign credit ratings. Increasing the granularity of information conveyed through sovereign credit ratings from their current single score (e.g., AAA to C-) to incorporate modular assessments of underlying risk factors through a modular sovereign credit rating could support a more risk-informed approach, by splitting out the contributions from different risk factors. Modular credit ratings would help ensure that the link between project-level and sovereign credit ratings can be more suitably assessed, allowing investors to evaluate the most applicable sovereign-level factors that could affect the project default (whereas currently all factors and biases within sovereign ratings contribute to the “sovereign ceiling” regardless)^79^. For example, the overall debt burden and debt-to-GDP ratio of the sovereign do not directly affect a privately-owned and -financed renewables project’s risk profile, but are important factors in sovereign credit ratings. Improved project-level data availability may also be essential to informing more risk-reflective behaviour from international investors, alongside scaling domestic capital markets and improving enabling environments for investment. Successful initiatives for the former have included the development of micro-finance institutions and other forms of innovative financing for renewables. For example, local currency loans and the “pay-as-you-go” model provided by Sun King have enabled over 27 m solar products to be distributed across Africa (with support from concessional loans from the IFC)^81^.

Domestic measures to improve the enabling environment for investment at a country level and reforms of the global financial architecture face many economic and geopolitical challenges. In the short term, support from international public financiers (IPFs) is essential to effectively de-risk clean energy projects and “crowd in” the private investment required, both by serving as an anchor point and addressing underlying risk factors^82^. Support mechanisms can include guarantee programmes^83^, strengthening national regulatory frameworks^28^ and a combination of capacity building and technical assistance^84^. Examples of successful interventions have included risk guarantees and political insurance for Ugandan hydroelectric power^85^, support for the development of bankable PPAs in Serbia^86^ and efforts to support enabling environments for private investment into clean energy by the World Bank^87^. IPFs can also provide finance at concessional rates^22^, which is key for LICs that we show here face some of the highest commercial costs of capital. Estimates show that concessional funding for clean energy investment will similarly need to scale up by five to six times, to ensure that clean energy investment in LMICs reaches a level that delivers on the Paris Agreement^53,88^. The New Collective Quantified Goal for climate finance, agreed at COP29^89^, included an ambition to expand international public and concessional financing. However, it  was criticised for lacking sufficient inclusion of grants and other below-market sources of financing^90^. Here, we show that Sub-Saharan Africa, Latin America and parts of Asia see high costs of capital that severely limit the feasibility of renewables and their deployment despite high mitigation potentials. International support in these regions would therefore be highly impactful to accelerating effective progress towards the UNFCCC target of tripling renewables and maximising the emissions reductions it delivers.

Whilst international flows of concessional climate finance are important to achieving the UNFCCC target, domestic interventions such as market reforms^19,91,92^, renewable energy subsidies^93^ and coordinated national energy planning^94^ are also needed. Tailored support which accounts for national contexts and circumstances^95^ will be important, for example, despite electricity costs from renewables falling rapidly over the last few decades^96^, capital expenditures per kilowatt of installed  capacity can still differ substantially across regions (up to nearly three times)^16^. Revenue stabilisation measures and auction processes for permits and support have also been shown to de-risk renewable investments^97^, whilst investment subsidies^93^ could be used to improve short-term viability in regions with high costs whilst local industry and workforces develop. Coordinating domestic policy measures and targets with the provision of international support, for example, through Just Energy Transition Partnerships^98^, can also play an important role in accelerating progress towards national and international targets, including tripling renewable capacity by 2030 and wider emissions targets under the Paris Agreement.

Despite the key role that the cost of capital plays in the economics of renewable technologies^35^, the regional approach used by many energy system models fails to capture the range of intraregional costs of capital, with typical regional groupings that are used containing countries at disparate stages and contexts to the energy transition^95,99,100^. Inaccurate assumptions for the cost of capital have been shown to substantially bias model results^36^, limiting the utility of energy research for policymakers. Here we have developed estimates of the cost of capital, renewable potential, LCOE-supply curves and lifetime mitigation globally at a high spatial granularity, which can serve as valuable inputs to future technoeconomic^46^ and energy system modelling efforts^68^, addressing data availability issues highlighted by other works^41,101^. Our results are also visualised online in an interactive webtool found at https://renewablescostninja.streamlit.app/, enabling easy exploration of the results under the impact of different deployment and financing cost assumptions. Using accurate, context-specific data is important to the usefulness of energy modelling, particularly in LMICs^102^, and the results from this work can help support academics, policymakers and industry stakeholders to ensure the accuracy and utility of technical insights from energy modelling studies. The use of context-specific cost of capital data is also valuable to the use of Integrated Assessment Models to explore scenarios for burden sharing and the equity implications of mitigation pathways, which will be an increasing focus under the IPCC’s AR7 workplan^103^. Further research efforts should be focused on developing a robust methodology for projecting the cost of capital forward, alongside broadening the coverage to emerging low-carbon technologies and accounting for subnational variance in the cost of capital for renewable firms^12^ and projects^26,104^ caused by project- and company-level risk factors not modelled here.

## Methods

### Renewable power profiles

Wind speed and solar irradiance data were sourced from NASA’s MERRA-2 dataset^105^, using a single representative year of data (2022). Wind speeds were converted into hourly wind turbine power output using the Virtual Wind Farm (VWF) model^51^, whilst solar PV power outputs were generated from the horizontal solar irradiance using Sandia Lab’s PVLib package^50^. Two heuristics were used to specify the solar PV panel orientation: 1) PV modules were orientated so as to face the equator, and 2) the tilt angle was calculated using Equation [1], from Bamisile et al^106^.:

1 $$\beta=3{\left|\varphi \right|}^{\frac{2}{3}}$$

where *β* is the tilt angle, and *φ* is the latitude, both in degrees.

### Modelling the cost of capital

Building on the approach used by IRENA^39^ and Calcaterra et al.^21^, the cost of capital for solar PV, onshore wind and offshore wind was estimated at a country level for 2026 using national data on country risk and renewable penetration. A full dataset of the cost of capital estimates using the same methodology presented here, which covers solar, wind and other energy technologies across the period 2015–2034, was recently published by Hatton et al.^27^. The cost of capital differs for equity and debt financing components, with equity financing typically requiring higher returns  as payments to equity investors are made last in the event of project failure. Weighting the costs of debt and equity investment by their proportions, as in Equation [2], gives the total cost of capital for a project (referred to here as the WACC) for a country (*x*) and technology (*t*). *C*_*E*_*, C*_*D*_ and *R*_*D*_ represent the cost of equity, cost of debt and debt share, respectively, with τ representing the corporate tax rate (as loan repayments are tax deductible in the majority of countries).

2 $${{WACC}}^{x,t}={R}_{D}^{x}{C}_{D}^{x,t}(1-{\tau }^{x})+(1-{R}_{D}^{x}){C}_{E}^{x,t}$$

The cost of debt and equity were calculated individually at the country and technology level, for 2026, using an adaptation of the approach set out by IRENA^39^ and used in Calcaterra et al^21^. with more detailed treatment of debt share in line with country risk. The cost of equity (*C*_*e*_) for a given country (*x*) and technology (*t*) was calculated using Equation [3], with contributions from the risk-free rate (*r*_*risk-free*_), country risk premium (*r*_*country*_), technology risk premium (*r*_*tech*_) and the equity risk premium (*r*_*equity*_). The latter accounts for the risk that equity investors take in being paid last in the event of project failure. The cost of debt (*C*_*D*_) for a given country (*x*) and technology (*t*) was calculated using Equation [4], which includes the same risk-free rate and technology risks but includes a country default spread (${r}_{{cds}}^{x}$) and debt lenders' margin (*r*_*debt*_). The lender's margin represents both returns for the debt issuer as well as additional margins to account for the technology- or project-specific risks that the lender bears when financing an infrastructure project, and so includes a portion of the technology risk where applicable. Both the cost of debt and the cost of equity were calculated for 2026. For countries without a reported country risk premium, their country risk was set to the highest reported value in their region, given that these countries are regarded as un-investable (i.e. “non-investment grade speculative”) on international capital markets due to extremely high country risk factors^60^.

3 $${C}_{E}^{x,t}={r}_{{risk}-{free}}+{r}_{{country}}^{x}+{r}_{{tech}}^{x,t}+{r}_{{equity}}$$

4 $${C}_{D}^{x,t}={r}_{{risk}-{free}}+{r}_{{cds}}^{x}+{r}_{{tech}}^{x,t}+{r}_{{debt}}$$

The risk-free rate was taken here as the US Federal Reserve’s interest rate on a 10-year government bond^107^, as US Treasury bonds are regarded as one of the safest investor assets and are typically used as a pricing benchmark. The country risk premium (${r}_{{country}}^{x}$), country default spreads (${r}_{{cds}}^{x}$) and equity risk premiums $({r}_{{equity}})$ for 2026 were extracted from Damodaran’s annual estimates of financial parameters^25^, in line with Calcaterra et al^21^ and IRENA^39^. The debt lender's margin (*r*_*debt*_) was taken as 2% in line with IRENA’s assumptions for large-scale infrastructure projects^39^, with its inclusion adjusted to avoid double-counting of technology risk by only adding the technology risk to the degree it exceeds the lender's margin. Tax rates (τ) were taken as national corporate tax rates using the Tax Foundation’s database^108^.

The technology premium (${r}_{{tech}}^{x,t}$) was evaluated based on the maturity of each technology for a given country in 2026, calculated using annual generation capacity statistics taken from Ember Climate^63^. Countries were categorised into Mature, Intermediate and Emerging markets for each technology, with technology-specific premiums and boundaries for market maturity based on penetration. Mature markets for solar PV and onshore wind were categorised as over 10% penetration, whilst for offshore wind, this was taken as 6%. For Intermediate markets, the lower boundaries were 5% and 3% respectively, whilst all other countries were categorised as emerging markets in line with IRENA^39^. Technology premiums ranged from 1.5 to 3.25% for onshore wind and solar PV inversely with maturity, whilst for offshore wind the technology premium was assumed to range from 2.6 to 4.35%^39^. To avoid stepwise changes in the technology risk premium at the boundaries of categorisations, the premium for Intermediate countries was calculated via linear interpolation using grid penetration, between the Mature-Intermediate boundary (where the tech premium is 3.25% for solar) and the Intermediate-Emerging boundary (where the tech premium is 1.5% for solar).

The debt share (R_D_) was assumed to vary inversely between 40% and 80% in a linear fashion with country risk premium, with low-risk countries assumed to have higher debt shares^39^ whilst country risk premiums were taken from Damodaran^25^. The boundaries of 40–80% were informed by the reported ranges in the Diacore and AURES2 projects for the EU-27, which ranged between 40 and 80% for solar PV and onshore wind^109,110^ (excluding results from Portugal and Spain, where some projects were financed with 100% equity through corporate financing structures). Notably, this improves on the approach used by IRENA^39^, which models a debt share of either 60%, 70% or 80% depending on the maturity of the technology in each market (without relating these assumptions to country risk premiums). Tax rates (${\tau }^{x}$) at a national level were extracted from reporting on corporate tax rates from the Tax Foundation^108^. Whilst many countries include a range of industry-specific tax incentives that may affect the overall cost of capital for renewables, due to the lack of data comparing these interventions across countries, the tax rate was assumed to be the same as the standard corporate tax rate in each jurisdiction. We note that renewable-specific tax rates may lead to our estimated costs of capital being higher based on these limitations, given that some countries provide preferential taxation rates or other fiscal incentives for clean energy projects to encourage investment.

### Levelised cost of electricity

The levelised cost of electricity (*LCOE*) is a common metric used to compare the economic viability of electricity production across different locations and/or sources^61^. It represents the price at which electricity would need to be sold to cover the costs of the project and give a net present value of zero. Equation [5] was used to calculate the LCOE, which requires input values for the capital expenditure (*CAPEX*), annual operating expenditure (*OPEX*) and electricity production (*E*_*t*_) for each year (*t*). The costs and electricity production in each year are then discounted using a discount rate (*r*), up to the plant’s assumed economic lifetime of 20 years (*n*). The country- and technology-specific WACC values estimated in this work using Equation [5] were used for the discount rate, reflecting the estimated financing terms for renewable projects.

5 $${LCOE}=\frac{{\sum }_{t}^{n}\frac{{{CAPEX}}_{t}+{{OPEX}}_{t}}{({1+r}){^t}}}{{\sum }_{t}^{n}\frac{{E}_{t}}{{\left(1+r\right)}^{t}}}$$

Capital costs for solar PV and onshore wind were taken from IRENA’s Renewable Generation Costs in 2025 report and applied at a regional level using regional weighted average deployment costs^16^ (with national costs used where available), with benchmarking also conducted against the GNESTE database^101^. For offshore wind, the global weighted average cost of US$2852/kW was used in all locations^16^. Capital costs for onshore wind are available at a regional level with some national deployment costs reported, whilst capital costs for solar PV are provided by IRENA for the 30 largest markets and by region. The weighted average for each region was extracted and used for countries in regions without national cost data, whilst for regions where deployment costs were not reported, the global weighted average cost of US$691/kW for solar and US$1041/kW for onshore wind were used^16^ (see Supplementary for full details at a country level). Annual operating costs for solar PV, onshore and offshore wind were taken as 2.3%, 2.6% and 3% of the initial capital investment, respectively, using the same assumptions used by the International Energy Agency^111^. All costs were converted to 2025 dollar terms using data on global inflation in 2024–2025 taken from the IMF’s World Economic Outlook (5.7%)^112^.

### Technical potential

To generate national and regional LCOE-supply curves, the technical renewable potential at each grid point of resolution (0.5 latitude by 0.625 longitude) modelled in the MERRA-2 meteorological data^105^ was evaluated in this work. The packing density for solar PV was taken to be 32.95 MW/km^2^, based on NREL’s estimates for fixed-axis utility-scale generation^113^. For onshore wind, a packing density of 6.52 MW/km^2^ was taken from Bosch et al^114^. Finally, for offshore wind, a packing density of 4 MW/km^2^ was extracted from NREL’s evaluation of capacity density considerations for offshore wind^115^.

The production potential at each location also depends on the available land that is suitable for renewable power, which may be affected by economic, technical or environmental constraints. Land use data was extracted using the GlobCover dataset^116^, which is generated from satellite data and categorises land use into 23 classes at a high spatial resolution. Corresponding land availability factors were extracted from Dupont et al.^117^ and Dupont et al.^118^ (see Supplementary) and used as a constraint to the annual production potential and maximum installed capacity at each location. Land use availability factors of 100% were assumed for offshore locations.

### Cost increase in meeting the COP28 tripling renewables target

Evaluating the potential increase in costs to meet the COP28 tripling target caused by high costs of capital in LMICs is challenging, due to uncertainties around a) regional distribution of new capacity, b) packing density of deployed capacity and c) non-technical factors that influence deployment (e.g., grid capacity, conflicting land uses, distance from demand centres, etc.). To give an upper bound for how high costs of capital in LMICs increase the annual costs of meeting the target, the following steps were undertaken: (1) locations in high-income countries were removed for each technology; (2) the lowest cost locations to meet the 5000 GW target were extracted, based on an assumption of 1 MW deployed at each location (i.e. 5m locations selected); (3) the average cost increase per year is calculated, across each location, using Equation [6]; and (4) the average cost increase was summed across the 5m locations in LMICs across the world.

6 $${Average}\, {cost}\, {increase}=\frac{{\sum}_{t}^{n} E_{t}*(LCOE_{national}- LCOE_{uniform})}{n}$$

### Renewable mitigation potential

Assessing lifetime renewable mitigation potential requires making assumptions around the trajectory of future grid intensities at a national level. Using Equation [7], the total mitigation over the project lifetime (n) was calculated using the annual technical electricity potential (${E}_{t}$) at each grid cell and the national grid carbon intensity (${{GI}}_{t}$), based on an assumption that each MWh of electricity generated from the solar or wind farm displaces a MWh of existing grid electricity. Initial grid intensities for the first year were taken from Ember Climate^63^ for 2025, with backfilling to the most recent year of data for countries without reported values for 2025. Grid intensity was modelled as falling linearly to zero over the 20-year project lifetime, i.e. that global average grid intensity reaches zero by 2045 (in line with the Net Zero Scenario from the International Energy Agency^3^). This was taken to be the case in both LMICs and high-income countries, though high-income countries are modelled to reach zero grid emissions intensities by approximately 2035 under the Net Zero Emissions scenario^3^. Lifetime carbon mitigation potential estimates developed here will therefore be larger in high-income countries compared to a scenario where they reach zero grid carbon intensities in 2035.

7 $${Abatement}=\,{\sum }_{t}^{n}{E}_{t}*{{GI}}_{t}$$

To estimate total mitigation potential for each country, data on total annual electricity generation in 2025 was also sourced from Ember Climate, with backfilling to the most recent year of data to fill in national data gaps^63^. The technical potential (${E}_{t}$) for each region was limited to the total annual generation in 2025, with the lowest cost locations used to create the cost-mitigation supply curves. To calculate the mitigation potential at each grid cell, 10% of the technical potential at each grid cell was assumed to be used to support the decarbonisation of national power grids, reflecting conflicting demands for land and other limits to infrastructure deployment, e.g., grid connections.

## Supplementary information

Supplementary Information

Transparent Peer Review file

Extended Data Figures

## Data Availability

The processed data generated in this study have been deposited in a Zenodo repository which can be found at: https://doi.org/10.5281/zenodo.17798379. An interactive webtool that allows visualisation of the data generated and exploration of the impact of differing cost assumptions has also been developed and is available at https://renewablescostninja.streamlit.app/.

## Code availability

The Python scripts for data processing and visualisation can be accessed in the GitHub repository, found at https://github.com/LukeHatton21/CostofCapitalLCOEModel (https://doi.org/10.5281/zenodo.20615929).
