# Supplementary material for: High costs of capital shape the mitigation effects of renewable energy deployment globally
Source: Nat Commun. 2026 Aug 13;17:9733. doi: 10.1038/s41467-026-75666-6 (PMC13562596; doi:10.1038/s41467-026-75666-6)
Supplement: Supplementary file 1 [file 41467_2026_75666_MOESM1_ESM.pdf]

Supplementary Information for

High costs of capital shape the mitigation effects of renewable energy deployment globally

Luke Hatton[1,2*], Gbemi Oluleye[3], Malte Jansen[4], Iain Staffell[3], Adam Hawkes[1]

[1] Department of Chemical Engineering, Imperial College London, London, United Kingdom
[2] Columbia Centre on Sustainable Investment, Columbia University, New York, United States
[2] Centre for Environmental Policy, Imperial College London, London, United Kingdom
[3] Science Policy Research Unit, University of Sussex, Brighton, United Kingdom
* Corresponding author: l.hatton23@imperial.ac.uk

Table of Contents

Supplementary Figure 1. 2
Supplementary Figure 2. 3
Supplementary Figure 3. 4
Supplementary Figure 4. 5
Supplementary Figure 5. 6
Supplementary Figure 6. 6
Supplementary Figure 7. 7
Supplementary Figure 8 8
Supplementary Table 1. 9
Supplementary Table 2. 10
Supplementary Table 3. 11
Supplementary Table 4. 12
Supplementary Table 5. 13
Supplementary References 14

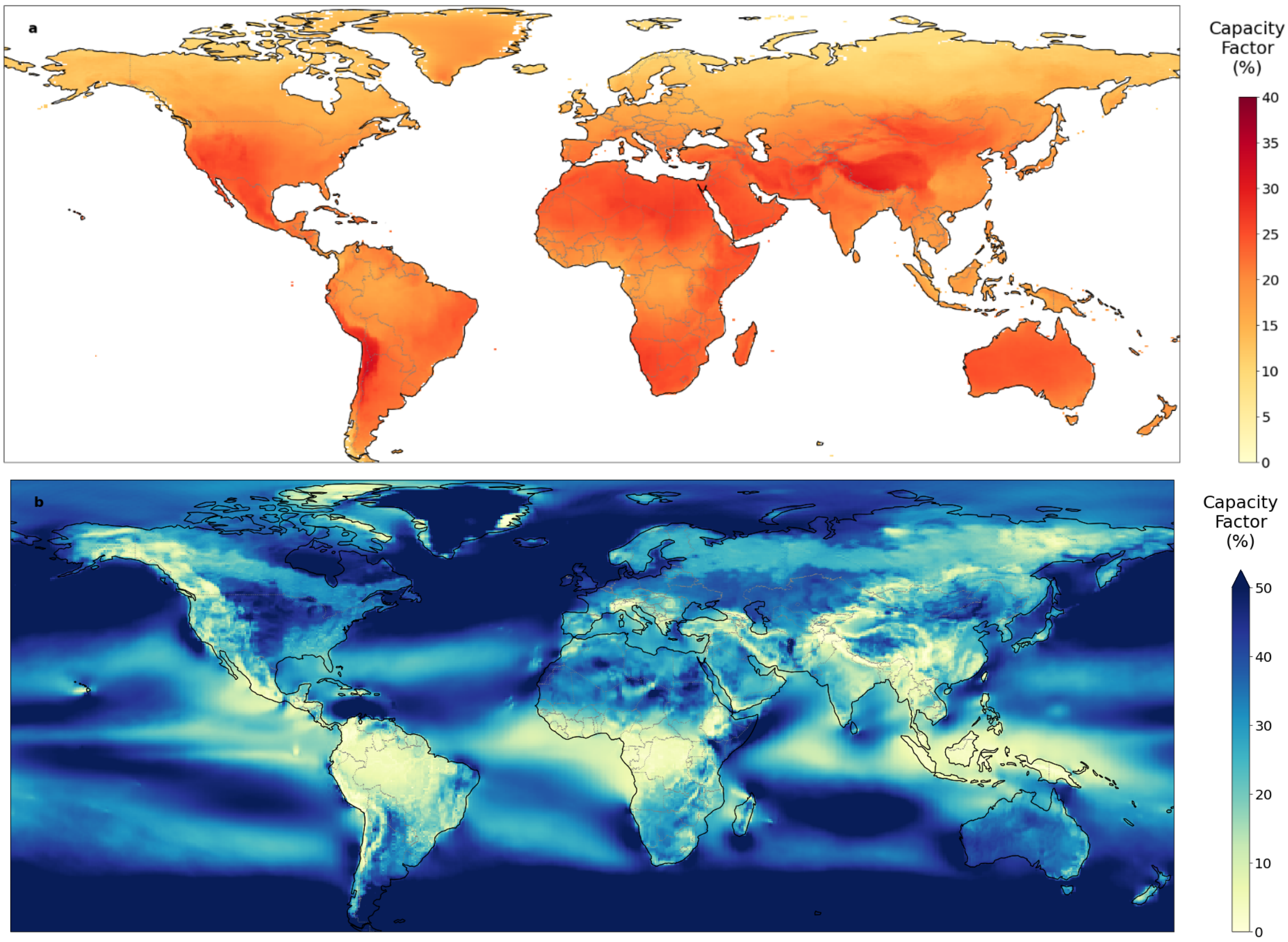

Supplementary Figure 1: The annual average capacity factor for renewable energy generation. *Capacity factor is displayed in % for a) solar PV and b) wind energy and are generated using the Renewables.Ninja method[1] and the PV Lib Python module developed by Sandia Labs[2]. The weather data used to generate wind and solar profiles was taken from NASA's MERRA-2 dataset[3] for a single representative year.*

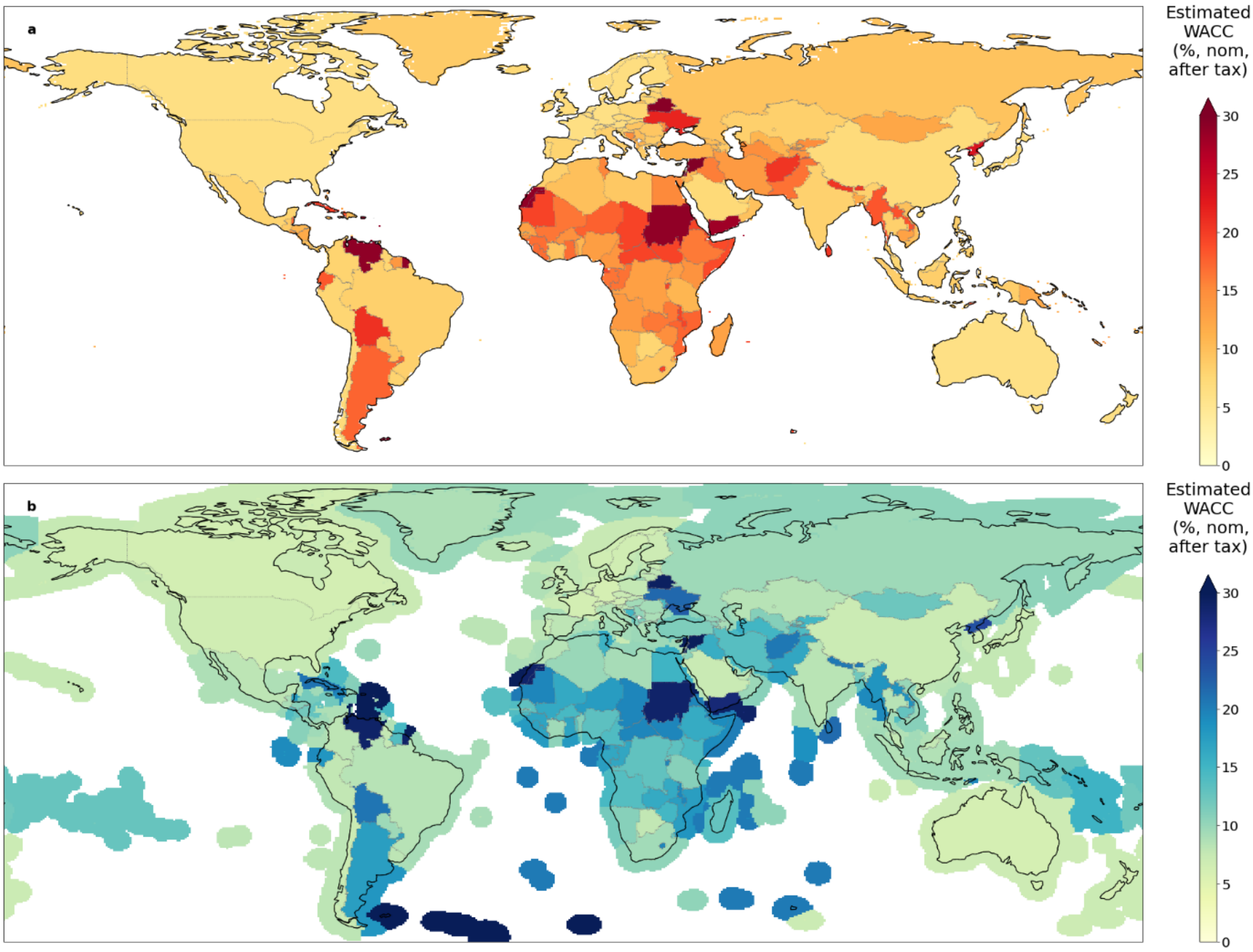

Supplementary Figure 2: Estimates of the nominal weighted average cost of capital (WACC) for 2026, mapped globally, for a) Solar PV, and b) Wind power. *Results are shown on a country-level basis for the 176 countries that had data on country risk premiums available from Damodaran[4], with country gaps filled by setting the national cost of capital to the maximum estimated in the country's region. The WACC was estimated by disaggregating contributions to the cost of debt and equity, advancing on the approach developed by IRENA[5] (see Methods). The full dataset using the same estimation approach for 10 electricity generation technologies has been published in Hatton et al. 2025[6], with short term projections to 2030 based on expected interest base rate changes.*

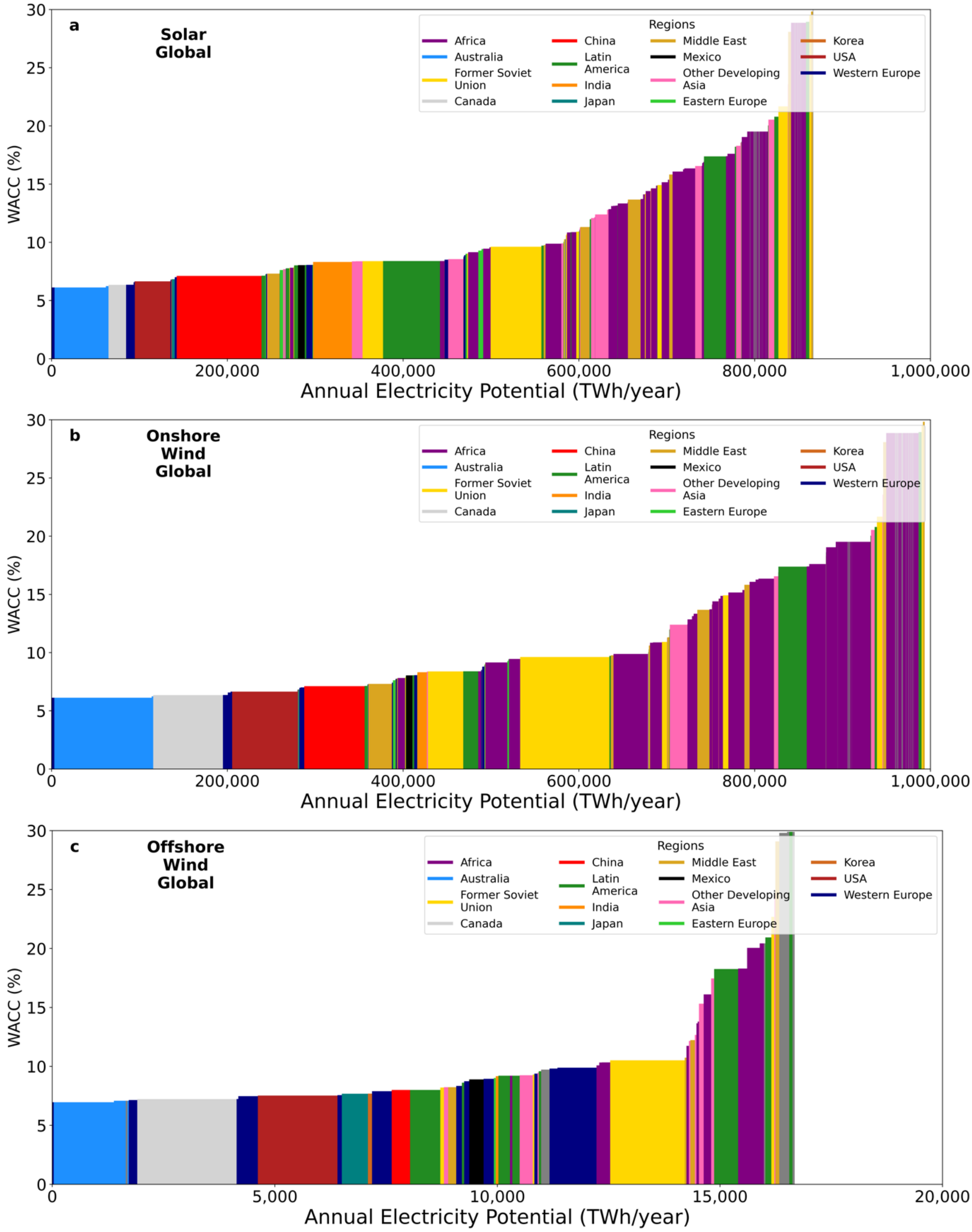

Supplementary Figure 3: Global technical potential curves for a) solar PV, b) onshore wind and c) offshore wind, ordered by estimated technology and country-specific cost of capital for 2026 and with regions highlighted. *Note the different scale used for offshore wind electricity potential. Packing densities of 32.95, 6.52 and 4.0 MW per km2 were used for solar, onshore wind and offshore wind, with land use constraints applied (see Supplementary). Minimum cut-offs of 10% and 18% average capacity factors across the year were applied to the solar and wind potentials to reflect constraints in economic feasibility of renewables deployment[7].*

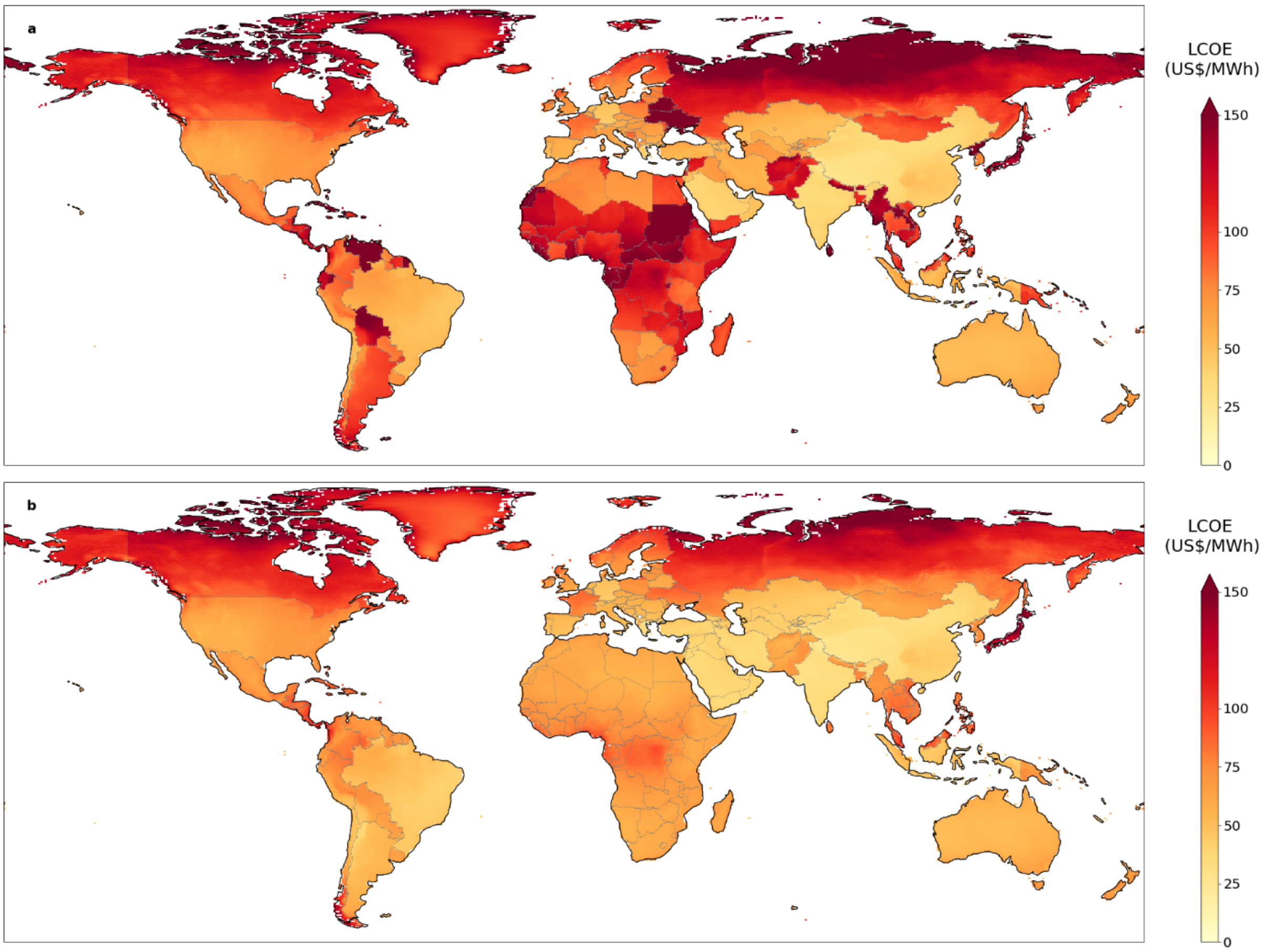

Supplementary Figure 4: Levelised cost of electricity from solar PV, plotted globally for a) country-specific costs of capital and b) a uniform nominal cost of capital of 7.1%. *The Uniform cost of capital was taken as the average estimated cost of capital for solar PV in the UNFCCC Annex II countries, which are commonly regarded as the high-income country grouping in climate negotiations (see Supplementary Table 2 for the full list of Annex II countries).*

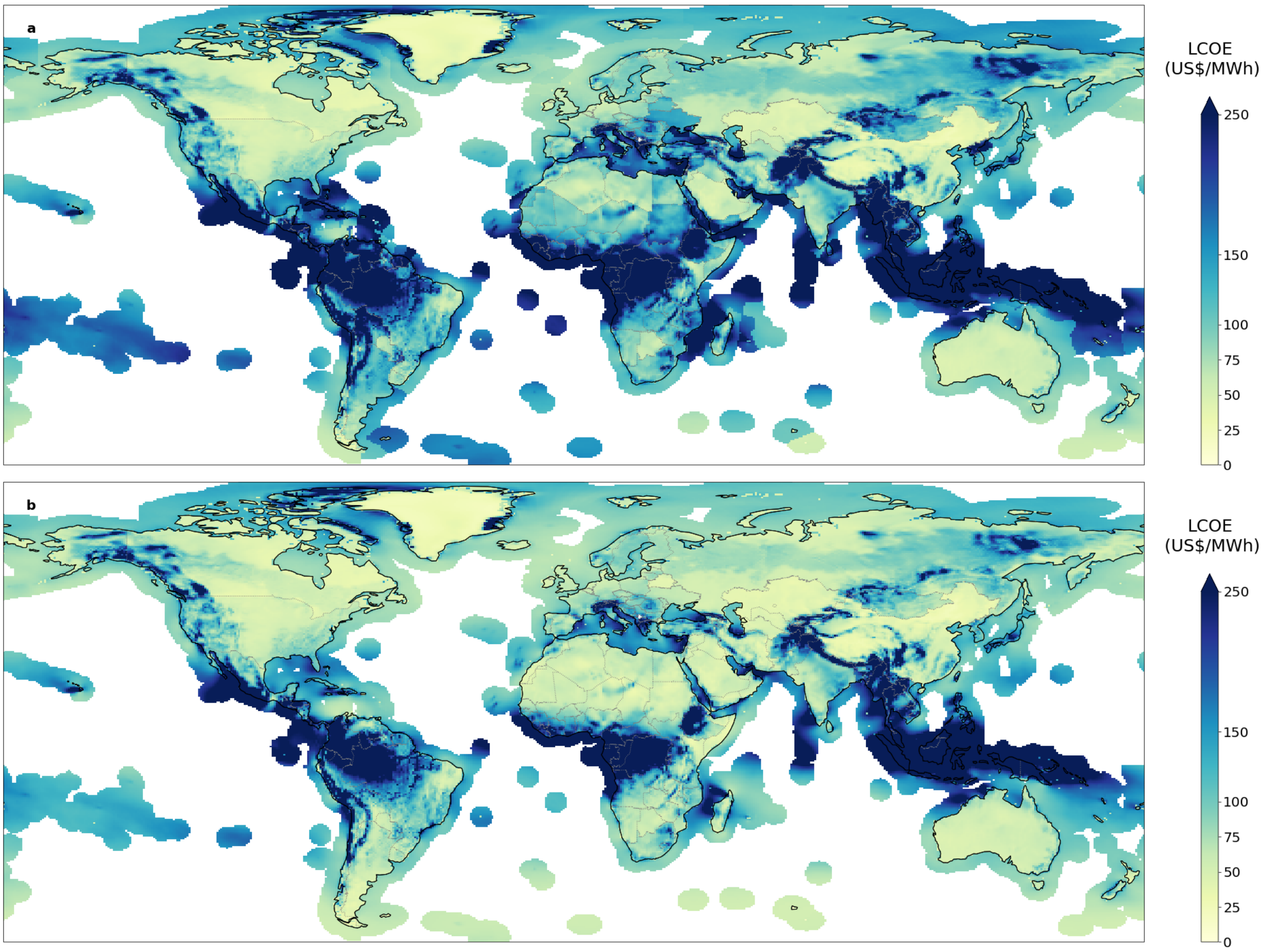

Supplementary Figure 5: Levelised cost of electricity from onshore and offshore wind, plotted globally using a) country-specific costs of capital and b) a uniform nominal cost of capital of 8.1% and 9.0% for onshore and offshore, respectively. *The Uniform costs of capital are taken as the average estimated cost of capital for onshore and offshore wind in the UNFCCC Annex II countries, which are commonly regarded as the high income country grouping (see Supplementary for the full list of Annex II countries).*

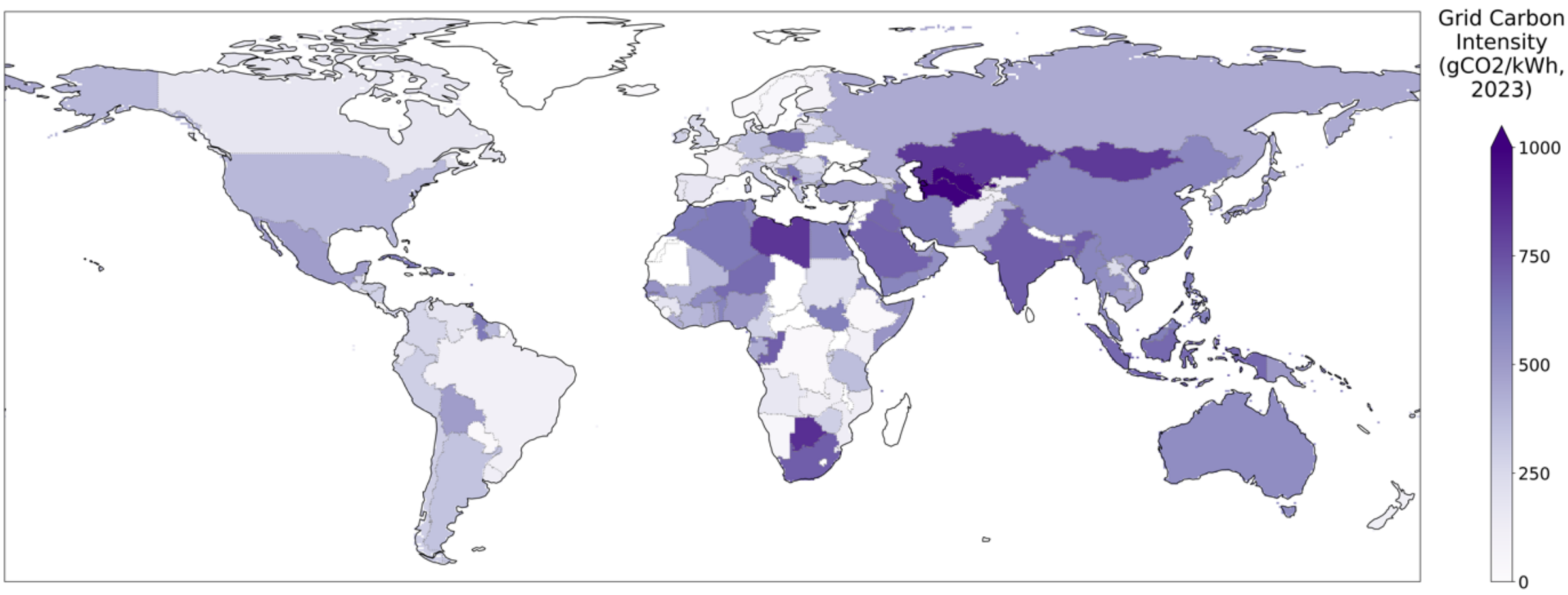

Supplementary Figure 6: Average grid electricity carbon intensity, plotted globally for 186 countries and/or regions. *Data is plotted in grams of carbon dioxide per kWh of generated electricity, taken from Ember Climate[8] for 2025, with missing countries filled in using data from 2021-2024 using the most recent data.*

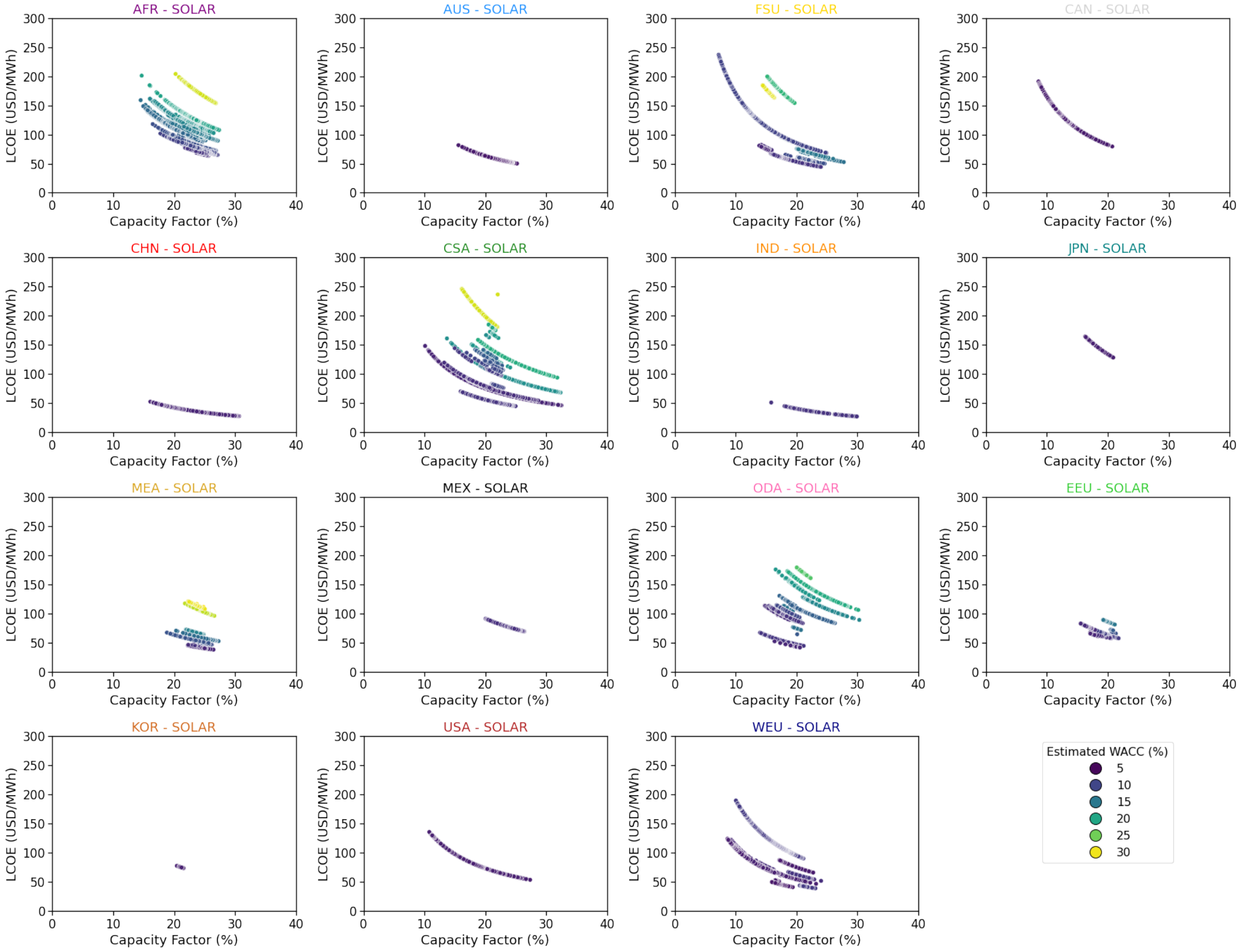

Supplementary Figure 7Relationships between capacity factor, estimated cost of capital and the evaluated levelised cost of electricity (LCOE), plotted for onshore and offshore wind for the major TIAM regions modelled in the study. *AFR: Africa, AUS: Australia and Oceania, FSU: Former Soviet Union, CAN: Canada, CHN: China, CSA: Central and South America, IND: India, JPN: Japan, MEA: Middle East, MEX: Mexico, ODA: Other Developing Asia, EEU: Eastern Europe, KOR: Korea, USA: United States of America, WEU: Western Europe.*

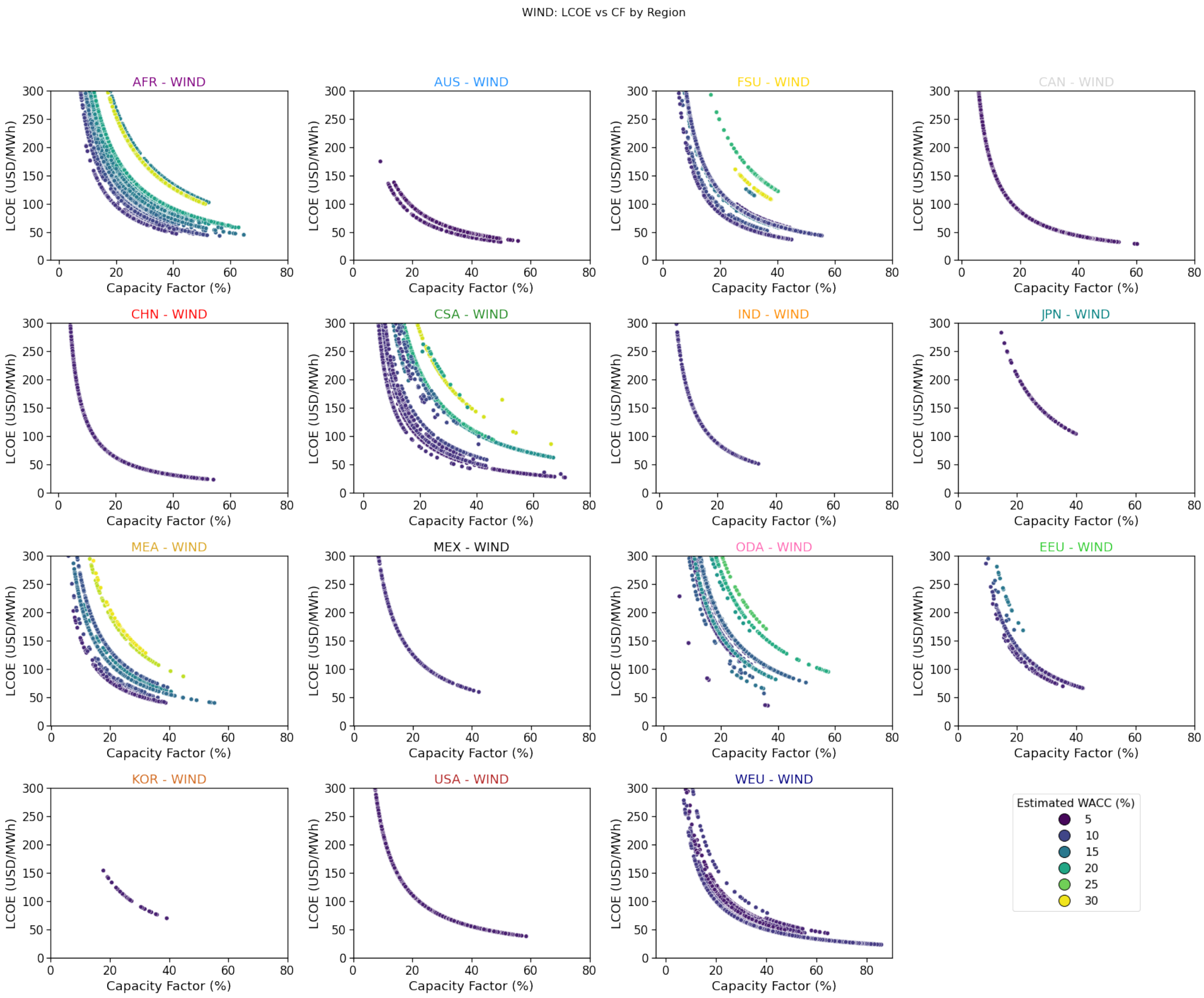

Supplementary Figure 8: Relationships between capacity factor, estimated cost of capital and the evaluated levelised cost of electricity (LCOE), plotted for onshore and offshore wind for the major TIAM regions modelled in the study*. AFR: Africa, AUS: Australia and Oceania, FSU: Former Soviet Union, CAN: Canada, CHN: China, CSA: Central and South America, IND: India, JPN: Japan, MEA: Middle East, MEX: Mexico, ODA: Other Developing Asia, EEU: Eastern Europe, KOR: Korea, USA: United States of America, WEU: Western Europe*

Region	Region Code	Countries
**Africa**	AFR	Algeria, Angola, Benin, Botswana, Burkina Faso, Burundi, Cameroon, Cape Verde, Central African Republic, Chad, Comoros, Congo, Congo Republic, Djibouti, Egypt, Equatorial Guinea, Eswatini (Swaziland), Ethiopia, Gabon, Gambia, Ghana, Guinea, Guinea-Bissau, Ivory Coast, Kenya, Lesotho, Liberia, Libya, Madagascar, Malawi, Mali, Mauritania, Mauritius, Morocco, Mozambique, Namibia, Niger, Nigeria, Reunion, Rwanda, Senegal, Seychelles, Sierra Leone, Somalia, South Africa, Sudan, Tanzania, Togo, Tunisia, Uganda, Zambia, Zimbabwe
**Australasia**	AUS	Australia, New Zealand
**Canada**	CAN	Canada
**China**	CHN	China
**Caribbean & South America**	CSA	Antigua and Barbuda, Argentina, Bahamas, Barbados, Belize, Bermuda, Bolivia, Brazil, Cayman Islands, Chile, Colombia, Costa Rica, Cuba, Dominica, Dominican Republic, Ecuador, El Salvador, French Guiana, Grenada, Guadeloupe, Guatemala, Guyana, Haiti, Honduras, Jamaica, Martinique, Netherlands Antilles, Nicaragua, Panama, Paraguay, Peru, St. Kitts and Nevis, St. Lucia, St. Vincent and Grenadines, Suriname, Trinidad-Tobago, Uruguay, Venezuela
**Eastern Europe**	EEU	Albania, Bosnia-Herzegovina, Bulgaria, Croatia, Czech Republic, Hungary, Montenegro, Poland, Romania, Slovakia, Slovenia
**Former Soviet Union**	FSU	Armenia, Azerbaijan, Belarus, Estonia, Georgia, Kazakhstan, Kyrgyzstan, Latvia, Lithuania, Moldova, Russia, Tajikistan, Turkmenistan, Ukraine, Uzbekistan
**India**	IND	India
**Japan**	JPN	Japan
**Middle East**	MEA	Bahrain, Cyprus, Iran, Iraq, Israel, Jordan, Kuwait, Lebanon, Oman, Qatar, Saudi Arabia, Syria, Turkey, United Arab Emirates, Yemen
**Mexico**	MEX	Mexico
**Other Developing Asia**	ODA	Afghanistan, Bangladesh, Bhutan, Brunei, Cambodia, Chinese Taipei, Cook Islands, Fiji, French Polynesia, Guam, Indonesia, Kiribati, Laos, Macau, Malaysia, Maldives, Mongolia, Myanmar, Nauru, Nepal, New Caledonia, Niue, North Korea, Pakistan, Papua-New Guinea, Philippines, Samoa, Singapore, Solomon Islands, Sri Lanka, Taiwan, Thailand, Vanuatu, Vietnam
**South Korea**	KOR	South Korea
**United States**	USA	United States
**Western Europe**	WEU	Austria, Belgium, Denmark, Finland, France, Germany, Gibraltar, Greece, Greenland, Iceland, Ireland, Italy, Liechtenstein, Luxembourg, Malta, Monaco, Netherlands, Norway, Portugal, San Marino, Spain, Sweden, Switzerland, United Kingdom, Vatican City

Supplementary Table 1: Country-region mappings used in the modelling of technical potential in this work. *Mappings were taken from the TIMES-Integrated Assessment Model, a global technology energy systems and climate bottom-up optimisation model that is use to explore global decarbonisation pathways.*

**Country**
Austria
Belgium
Canada
Denmark
Finland
France
Germany
Greece
Iceland
Ireland
Italy
Japan
Luxembourg
The Netherlands
New Zealand
Norway
Portugal
Spain
Sweden
Switzerland
Turkey
United Kingdom
United States of America

Supplementary Table 2: List of Annex II countries, as defined by the UNFCCC. *Annex II countries under the UNFCCC consistent of high income industrialised nations that are obligated to provide financial resources and technology transfer to low and middle-income countries to help tackle climate change.*

ID	GlobCover global legend	Merged land use category	Solar PV land use factor	Wind land use factor
150	Sparse (>15%) vegetation (woody vegetation, shrubs, grassland)	Barren	10%	90%
200	Bare areas	Barren	10%	90%
14	Rainfed croplands	Cropland	50%	60%
20	Mosaic Cropland (50-70%) / Vegetation (grassland, shrubland, forest) (20-50%)	Cropland	50%	60%
11	Post-flooding or irrigated croplands	Cropland natural	10%	70%
30	Mosaic Vegetation (grassland, shrubland, forest) (50-70%) / Cropland (20-50%)	Cropland natural	10%	70%
40	Closed to open (>15%) broad-leaved evergreen and/or semi-deciduous forest (>5m)	Forest	0%	10%
50	Closed (>40%) broad-leaved deciduous forest (>5m)	Forest	0%	10%
60	Open (15-40%) broad-leaved deciduous forest (>5m)	Forest	0%	10%
70	Closed (>40%) needle-leaved evergreen forest (>5m)	Forest	0%	10%
90	Open (15-40%) needle-leaved deciduous or evergreen forest (>5m)	Forest	0%	10%
100	Closed to open (>15%) mixed broad-leaved and needle-leaved forest (>5m)	Forest	0%	10%
120	Mosaic Grassland (50-70%) / Forest/Shrubland (20-50%)	Grassland	10%	90%
140	Closed to open (>15%) grassland	Grassland	10%	90%
230	No Data	No data	0%	0%
110	Mosaic Forest/Shrubland (50-70%) / Grassland (20-50%)	Shrubland	5%	50%
130	Closed to open (>15%) shrubland (<5m)	Shrubland	5%	50%
220	Permanent snow and ice	Snow and ice	0%	0%
190	Artificial surfaces and associated areas (urban areas >50%)	Urban	0%	100%
160	Closed (>40%) broad-leaved forest regularly flooded - Fresh water	Water	0%	100%
170	Closed (>40%) broad-leaved semi-deciduous and/or evergreen forest regularly flooded - Saline water	Water	0%	100%
180	Closed to open (>15%) vegetation (grassland, shrubland, woody vegetation) on regularly flooded or waterlogged soil - Fresh, brackish or saline water	Water	0%	100%
210	Water bodies	Water	0%	100%

Supplementary Table 3: Land use availability factors used to convert electricity production at each gridcell to annual technical potentials*. Land use factors were taken from Dupont et al 2020[9](solar) and Dupont et al[10] 2018 (wind). The underlying land use characterisations were taken from the GlobCover dataset[11].*

Region	Onshore Wind (2024 USD/kW)	Solar PV (2024 USD/kW)	Offshore Wind (2024 USD/kW)
Africa	1333	1093	2845
Central America & the Caribbean	2108	1375	2845
Eurasia	1425	1030	2845
Europe	1659	779	2845
Oceania	1363	944	2845
Other Asia	1912	1133	2845
Other North America	1238	1206	2845
Other South America	1492	1048	2845
Middle East	1041	691	2845
Other	1041	691	2845

Supplementary Table 4: Regional deployment costs for solar PV, onshore wind and offshore wind. *Costs were taken from IRENA's Renewable Power Generation 2024 report*[12]*. All costs were converted to 2025 USD dollar terms using the global inflation in 2024 of 5.7% based off of the IMF's World Economic Outlook 2025*[13] *due to IRENA's Renewable Power Generation Costs in 2025 not being published at the time of final submission. Full regional-country mappings used by IRENA can be found in IRENA's Renewable Power Generation 2024 report*[12]*.*

Country	Region	Onshore Wind Capex (2024 USD/kW)	Solar PV Capex (2024 USD/kW)
Argentina	Other South America	1669	895
Austria	Europe	1702	
Australia	Oceania	1381	944
Brazil	Brazil	1307	724
Canada	North America	1238	1213
Chile	Other South America	1313	
China	China	856	591
Germany	Europe	1670	593
Dominican Republic	Central America & the Caribbean	2108	
Egypt	Africa	1435	
Spain	Europe	1399	822
Ethiopia	Africa	2273	
Finland	Europe	1424	
France	Europe	1860	1104
United Kingdom	Europe	1926	
Greece	Europe	1967	566
China	China	856	591
Croatia	Europe	1559	
Ireland	Europe	1605	
India	India	1110	525
Italy	Europe	1408	804
Japan	Other Asia	2891	1911
Morocco	Africa	1216	
China	China	856	591
Mexico	Other North America	1614	
Netherlands	Europe	1685	641
New Zealand	Oceania	1162	
Peru	Other South America	1065	
Philippines	Other Asia	2325	
Pakistan	Other Asia	1321	
Poland	Europe	1838	872
Sweden	Europe	1773	
Turkey	Middle East	1424	
China	China	856	591
Ukraine	Eurasia	1642	
United States	North America	1568	1058
South Africa	Africa	1773	

Supplementary Table 5: National deployment costs, for solar PV and onshore wind. *National deployment costs were taken from IRENA's Renewable Power Generation Costs in 2024 report*[12]*. Where national deployment costs were not available, regional deployment costs were used instead. All costs were converted to 2025 USD dollar terms using the global inflation in 2024 of 5.7% based off of the IMF's World Economic Outlook 2025*[13] *due to IRENA's Renewable Power Generation Costs in 2025 not being published at the time of final submission..*

Supplementary References

1. Staffell, I. & Pfenninger, S. Using bias-corrected reanalysis to simulate current and future wind power output. *Energy* **114**, 1224–1239 (2016).
2. F. Holmgren, W., W. Hansen, C. & A. Mikofski, M. pvlib python: a python package for modeling solar energy systems. *JOSS* **3**, 884 (2018).
3. Gelaro, R. *et al.* The Modern-Era Retrospective Analysis for Research and Applications, Version 2 (MERRA-2). *J. Climate* **30**, 5419–5454 (2017).
4. Aswath Damodaran. Country Default Spreads and Risk Premiums. https://pages.stern.nyu.edu/~adamodar/New_Home_Page/datafile/ctryprem.html (2024).
5. IRENA. *The Cost of Financing for Renewable Power*. https://www.irena.org/Publications/2023/May/The-cost-of-financing-for-renewable-power (2023).
6. Hatton, L., Staffell, I., Jansen, M., Oluleye, G. & Hawkes, A. Historical and future projected costs of capital for ten energy technologies across 176 countries. Zenodo https://doi.org/10.5281/ZENODO.15227849 (2025).
7. Chu, C.-T. & Hawkes, A. D. A geographic information system-based global variable renewable potential assessment using spatially resolved simulation. *Energy* **193**, 116630 (2020).
8. Ember Climate. Yearly Electricity Data. https://ember-energy.org/data/yearly-electricity-data/.
9. Dupont, E., Koppelaar, R. & Jeanmart, H. Global available solar energy under physical and energy return on investment constraints. *Applied Energy* **257**, 113968 (2020).
10. Dupont, E., Koppelaar, R. & Jeanmart, H. Global available wind energy with physical and energy return on investment constraints. *Applied Energy* **209**, 322–338 (2018).
11. Bontemps, S. *et al.* GLOBCOVER 2009. (2011).
12. IRENA. Renewable Power Generation Costs in 2024. (2025).
13. IMF. *World Economic Outlook 2025*. https://www.imf.org/en/Publications/WEO/Issues/2025/04/22/world-economic-outlook-april-2025 (2025).
