# Supplementary material for: High costs of capital shape the mitigation effects of renewable energy deployment globally
Source: Nat Commun. 2026 Aug 13;17:9733. doi: 10.1038/s41467-026-75666-6 (PMC13562596; doi:10.1038/s41467-026-75666-6)
Supplement: Supplementary file 2 [file 41467_2026_75666_MOESM2_ESM.pdf]

nature portfolio

Peer Review File

High costs of capital shape the mitigation effects of renewable energy deployment globally

Corresponding Author: Mr Luke Hatton

This manuscript has been previously reviewed at another journal. This document only contains information relating to versions considered at Nature Communications.

This file contains all reviewer reports in order by version, followed by all author rebuttals in order by version.

Version 0:

Reviewer comments:

Reviewer #1

(Remarks to the Author)
This paper addresses an important, relevant, and timely topic. The introduction provides a clear explanation of the problem and its significance.

However, the manuscript would benefit from a more explicit discussion of its main contributions relative to the existing literature. Although the dataset provided is highly valuable and will likely be widely used, the analysis presented is limited in scope, particularly with regard to the scenario design and scenario choices.

The authors refer to COP28 targets but do not place their scenarios in the context of these goals or other relevant emissions targets. Despite this limitation, the effort remains a useful contribution and the database itself represents a valuable resource for the community. However, the text tends to be overly descriptive and could be significantly improved in terms of structure and engagement, especially if the scenario analyses became more meaningful.

Specific comments:

Lines 58–64, page 2: The distinction between points (1), (3), and (4) is unclear. Please clarify how these are conceptually different.How one is sure that 3 and are not included in 1?

Line 65: High-income countries also provide fossil fuel subsidies. Please revise to reflect this nuance.

Lines 104–105: “Africa” is repeated twice—please correct.

Line 111: Please rephrase for clarity. “MW” refers to capacity, not to electricity or energy per se.

Figure E: Panels are labeled “c” and “d” but should be “a” and “b”.

Lines 222–223: The 5% increase is not clearly explained. Is this uniformly distributed? Please specify how this numbers were derived and applied.

Scenario design: The comparison between uniform and national scenarios lacks a clear narrative. It is difficult to understand the logic or policy relevance behind the scenario choices and what message the authors intend to convey.

Figures: There appear to be two different “Figure 3”s. Please fix this inconsistency.

Throughout the text: Some repetitions could be removed for clarity and conciseness. Consider tightening the writing to improve readability and flow.

The Work offers a useful data resource, the analytical framing and textual clarity require further refinement to fully realize its

potential contribution.

(Remarks on code availability)
i did not review the code

Reviewer #2

(Remarks to the Author)
The paper estimates the impact that national cost of capital values have on LCOE and subsequently on the carbon abatement potential. It argues that developing countries have higher costs of capital, which in turn increases the LCOE in comparison to the uniform cost of capital (CoC) scenario. As developing countries have the highest CoC and among the highest renewable energy potential (especially solar), a lot of renewables capacity additions are being held back. The high CoC hinders project development in those countries, compared to developed and industrialized countries (like China).

The paper is of high quality, and it is very well written and structured. The images are compelling and easy to understand, with some exceptions (see below). The paper also cites all the relevant literature. Nevertheless, I still have some comments for the authors:

Major comments
1. The first main result (starting in line 205) is already known to the field. Differences in costs of capital obscure LCOE levels, making them lower in developed and higher in developing countries. Hence, this is not such a major novelty. I wonder if this is really needed in the main body of the text. If the aim is to present this paper to wide audiences that are not familiar with the subject, then yes. If the target group are experts in the field, than no. In addition, Figure 1 seems to repeat itself three times.
2. Connected to this, the main novelty I see in Figure 1 is that the LCOE are calculated on a very granular level. This is an interesting methodological contribution; however, I wonder why is it needed? The macroeconomic risk which drives the CoC is the same for the entire country. Hence why show LCOE in higher resolution? I would ask the authors to better explain the relevance of this granular approach. Figure 1 seems to be different mainly along the country lines anyway. Probably there are LCOE differences within the country, because of the different solar and wind potential distribution, but this is not seen from the map because of the global focus.
3. In my view the main contribution of the paper is that high CoC slow down the uptake of renewables in countries where the abatement potential is the highest. I did not yet see someone making this connection. However, this result is presented only later in the results section, although I believe it's the most relevant one. I would urge the authors to give this result more prominence in the text. The authors do mention the abatement potentials in the intro. However, it took me until the results section to finally understand why the authors are calculating the abatement potentials. It needs to be better explained in the intro. The connection between CoC, LCOE and abatement potential is not clear.
4. The authors suggest in the discussion that the costs of capital have to come down in developing countries. Since the CoC are mainly driven by country risk, how do the authors suggest to achieve this? The current suggestions sound a bit superficial. Concessional finance enables some projects to reduce the CoC. Its mainly arranged for projects that open a market – for instance, the first onshore wind farm in the country etc. Apart from this, there are good examples where multilateral finance institutions helped drive down risk. For instance, the World Bank in Serbia and its impact on making a bankable PPA, which has driven the domestic wind energy market. But this finance source / assistance will not be available to most of the renewable energy potential that needs to be developed. Small scale solar PV would have to rely on commercial banks. How should the small investors in Bangladesh tap into the concessional finance grants etc. and assistance? Are there some loan programs for microloans for solar PV? I would ask the authors to be more specific about measures to reduce the cost of capital, perhaps by giving some more specific examples.

Minor comments
1. In Figure and Figure it would be useful to have a list of abbreviations for the regions. Perhaps a table in the Appendix listing the countries within one region.
2. The authors use post tax WACC. The WACC rates here depend also on the country tax rates. Hence, the final WACC values used could be affected by the country's taxation rules. I think that post tax WACC can still be used as the authors are probably looking at the private economic perspective. However, I think it would be useful to add this as a note in the text.
3. The debt ratio values are now in a range that is informed by the Diacore project. I would like to highlight to the authors that there were newer studies like Diacore, such as the AURES2 cost of capital survey for Europe.
4. I would restructure the into so that the main purpose statement of the paper comes much sooner. Now it comes in paragraph 9. It should come the latest until paragraph 4. I would urge the authors to come to the point much sooner.

(Remarks on code availability)

Reviewer #3

(Remarks to the Author)
1. The paper makes an important contribution in terms of raising the issue of high cost of capital in developing countries as an important barrier to upscaled implementation of RE and supporting the argument with empirical evidence.

2. While there are data gaps for some country/region level data points, the overall point is well supported and availability of the code would enable researchers to replicate and test results in future.

3. Discussion section: Lines 436-437: The point about using accurate, context specific data to enhance usefulness of energy modeling, especially in developing countries could be further strengthened by extending the use of such data also in global Integrated Assessment Models (IAMs) that seek to examine alternative scenarios for burden sharing and decarbonization across regions and countries while aligning with global cumulative GHG emission limits.

(Remarks on code availability)
Code has been made available along with README file, so we assume that the analysis is reproducible. However, I have not installed and run it to check this.

Version 1:

Reviewer comments:

Reviewer #1

(Remarks to the Author)
I think the authors have responded in a satisfactory way to the comments made.

(Remarks on code availability)

Reviewer #2

(Remarks to the Author)
1.Thank you for your revisions! This will be a fine paper. Still, I want to challenge you on one main account, and this relates to the novelty of your work. I can see that combining cost of capital, LCOE and grid intensities is novel as it enables us to see how crucial financial is for pushing renewables where they matter the most. Still, in your text you seem to put an equal amount of emphasis on the findings that LCOE is higher in developing countries when using non-uniform CoC etc. In my view this is not novel and it has been covered in the literature many times, as you also rightly indicate in the intro. This for instance relates to the finding from the abstract: "We show that high costs of capital 16 substan ally increase LCOEs in developing countries, adding an average of US$21 (solar) and 17 US$26 (onshore wind). Financing costs cons tute up to 78% of the LCOE, compared to 40–18 42% under uniform global costs of capital".

You write: "Combining the technical and economic analysis, we evaluate how much cost of capital raises the LCOE across over 96,000 grid points, the impact that this has on regional cost-supply curves and implications for national and regional progress towards the COP28 goal. We find that locations in Africa, Central Asia and South America would have levelised costs within or below US LCOE ranges, if countries could access developed countries' financing terms, with the share of financing costs in the overall LCOE rising from 40% to nearly 76%, an average increase in EMDEs of US$21/MWh (solar) and US$26/MWh (onshore wind)."

If you are Sudan and have points A and B on your grid, the difference between A and B in costs of capital will be the same. Then the LCOE difference comes from renewable energy potential and not cost of capital. Hence, I would again like to question if you should really stress this as the most important aspect of your paper. In summary: grid carbon intensity plus bigger potential in developing countries and the lack of using this if financing is not there, is in my view the main hook of the paper, whereas "look we model 96000 points" and show differences between developed and developing countries is not that novel in my view.

Connected to this, could you show if there are any intra country variations in your LCOE values that are driven by factors other than country risk. Seeing this variability would justify your granular approach, although, as stated earlier I would be surprised if you had large variations that are driven by CoC within a single country.

2.In the new paragraph in the discussion section (918 – 984 in the Track Changes version) you now discuss a very specific example, which I like and this seems to address my comment directly. In there you write: "These are informed by sovereign credit ratings and the "sovereign ceiling" effect69, which an increasing body of literature has shown to be subjective70,71 and structurally biased against EMDEs72, underlining the need for reform of the structure and methodologies of current credit rating agencies. Data on default rates and returns from infrastructure and energy projects in EMDEs also does not reflect levels implied by the ceiling of sovereign credit ratings73, demonstrating that it is largely the perception of higher risks that is dissuading investors. Improved project-level data availability will be essential to informing more risk-reflective".
After reading this for the first time, I could not understand the term sovereign ceiling effect and how this relates to subjective risk perception. I would appreciate it if you could clarify that link.

(Remarks on code availability)

Reviewer #3

(Remarks to the Author)
The comments have been duly addressed.
The paper may be accepted.

(Remarks on code availability)
-

Version 2:

Reviewer comments:

Reviewer #2

(Remarks to the Author)
Thanks for the revision. I have only few smaller comments:

1) To improve the readability of the introduction please separate the last paragraph into two, at the point where you write your purpose statement (line 94, page 3)

2) In the discussion section, you write: "Failure to bring down the cost of capital in EMDEs will mean that less developed countries, including Bangladesh, will be unable to access the economic benefits of falling renewable deployment costs" In this sentence, and similar once before, you are saying that the high costs of capital is causing a higher LCOE and this is stopping investments. However, the high CoC is the consequence of overall country instability. For instance, the issue that there is widespread corruption, and as a developed you might need to heavily rely on connections to push your project or will need to engage in corrupt activities to get your permits higher. I suggest that you incorporate this connection with the higher CoC more - investors are not investing because of higher CoC caused by the lack of basic economic functions like rule of law etc.

3) In line 442, page 17 you write "As corporate and project credit ratings are largely capped at the country's sovereign credit rating (a "sovereign ceiling" for private credit ratings which cannot typically be exceeded73), inaccuracies or misperceptions in sovereign risk can severely constrain project financing conditions. An increasing body of literature has demonstrated the subjectivity and distance bias in sovereign credit ratings for developing countries74,75, as well as structural biases in their methodology against EMDEs76, underlining the need for reform of the structure, methodologies and role of credit rating agencies to help reduce the cost of capital. For example, modular sovereign credit ratings could aid a more risk-informed approach by investors than attempting to reflect a wide range of risk factors into one score (some of which will be applicable to the economic performance of projects or corporates operating under a given sovereign)77".

Here: a) I am not sure I understand your text. You write that "project credit ratings are largely capped at the country's sovereign credit rating". I understand this as project financed having a base rate defined by the countries sovereign credit rating. Is this correct? Please clarify and improve text for better understanding. Further, did I understand if you are suggesting to uncouple the evaluation of individual projects from this sovereign credit rating? The sentence where you talk about distance bias seems to suggest that. Further, it is unclear what you mean by distance bias - please clarify. Also you write about a modular credit rating (which I assume is the decoupling of overall country risk from individual project risk). Please write in the text a proper definition of this newly introduced term. On that note I have one conceptual comments as well. So what risks would then not be evaluated in the "renewables modular credit rating"? Which risk is relevant for country risk and not relevant for renewables? I would assume that all country-related risks are important like: currency, rule of law, overall business environment etc.

4) You write in line 463 about a reform of the overall finance architecture. May I suggest to the authors to stick more to the country level discussion before discussing the global framework. For instance, it would be useful for project developers to have something like a one-stop-shop or preferential treatment for permits and basically local help when getting the project permits, which could help with corruption-related issues.

(Remarks on code availability)

Version 3:

Reviewer comments:

Reviewer #2

(Remarks to the Author)
No comments left!

(Remarks on code availability)
No comments left!

Open Access This Peer Review File is licensed under a Creative Commons Attribution 4.0 International License, which permits use, sharing, adaptation, distribution and reproduction in any medium or format, as long as you give appropriate credit to the original author(s) and the source, provide a link to the Creative Commons license, and indicate if changes were made.
In cases where reviewers are anonymous, credit should be given to 'Anonymous Referee' and the source.
The images or other third party material in this Peer Review File are included in the article's Creative Commons license, unless indicated otherwise in a credit line to the material. If material is not included in the article's Creative Commons license and your intended use is not permitted by statutory regulation or exceeds the permitted use, you will need to obtain permission directly from the copyright holder.
To view a copy of this license, visit https://creativecommons.org/licenses/by/4.0/

We thank the reviewers for their positive assessment and constructive comments that have helped us to improve this manuscript. We respond to each point below in blue and new text added to the manuscript is quoted *in italics*.

Reviewer #1

This paper addresses an important, relevant, and timely topic. The introduction provides a clear explanation of the problem and its significance.

> **Response:** We thank you for your valuable feedback and comments on our manuscript draft, and are glad that you agree that it is an important, relevant and timely topic. We have responded to all comments and made revisions based on all of them.

However, the manuscript would benefit from a more explicit discussion of its main contributions relative to the existing literature. Although the dataset provided is highly valuable and will likely be widely used, the analysis presented is limited in scope, particularly with regard to the scenario design and scenario choices.

> **Response:** We thank you for your feedback on how the manuscript could be improved, including through a more explicit discussion of the main contributions beyond the existing literature. We are glad that you think the dataset will be highly valuable and likely be widely used. We have strived in our response to expand the scope of the analysis in response to your and other reviewer's helpful feedback.
>
> We have included the following sentences into the introduction to explicitly discuss what it aims to contribute relative to the existing literature:
>
> *Marked differences in the geographic distribution of high quality renewable energy resources and accessible costs of capital for project developers are a key driver of the concentration of deployment, often out of line with locations that have the best renewable potential[43]. Whilst this holds implications for cost and equity of the global energy transition[22], the implications on the overall effectiveness of mitigation efforts and progress towards the COP28 goal have not yet been explored in depth.*
>
> *However, there has been limited examination of the interplays between renewable LCOEs, costs of capital and lifetime abatement potential of renewables in the literature. Here, using a global geospatial model, we present global estimates of the levelised cost of electricity (LCOE), annual technical potential and lifetime abatement of solar, onshore wind and offshore wind power at a high geospatial granularity, using country- and technology-specific costs of capital for over 150 countries in 2024. Combining the technical and economic analysis, we evaluate how much cost of capital raises the LCOE across over 96,000 grid points, the impact that this has on regional cost-supply curves and implications for national and regional progress towards the COP28 goal*
>
> As well as including explicitly its contributions relative to the existing literature in the final paragraph of the discussions:

> *Inaccurate assumptions over the cost of capital have been shown to substantially bias model results[34], limiting the utility of energy research for policymakers. Here we develop estimates of the cost of capital, renewable potential, LCOE-supply curves and lifetime abatement at a high spatial granularity, which will serve as valuable inputs to future technoeconomic[43] and energy system modelling efforts[62], addressing data availability issues highlighted by other works[39,93]. Using accurate, context-specific data is important to the usefulness of energy modelling, particularly in developing countries[94], and the results from this work will help support academics, policymakers and industry stakeholders to maximise the utility of technical insights from energy modelling tools*

The authors refer to COP28 targets but do not place their scenarios in the context of these goals or other relevant emissions targets. Despite this limitation, the effort remains a useful contribution and the database itself represents a valuable resource for the community.

> **Response:** We are glad to hear that you think that the effort is a useful contribution and that the database itself will be a valuable resource. Following the first round of reviews, we have also uploaded the underlying data and results to a Zenodo repository (https://zenodo.org/records/17798380) in recognition of the value that the data holds for other analyses.
>
> In response to your comment around placing our analysis in the context of the COP28 goals or other relevant emissions targets, we have included the following additions into the introduction:
>
> *Clean energy investment needs to rise globally to US$1.5 trillion per year between 2025-2030[1], if the world is to achieve the COP28 goal of tripling global renewable capacity by 2030 to at least 11,000 GW[2] and the Paris-aligned goal of net zero emissions by mid-century[3].*
>
> *Combining the technical and economic analysis, we evaluate how much cost of capital raises the LCOE across over 96,000 grid points, the impact that this has on regional cost-supply curves and implications for national and regional progress towards the COP28 goal.*
>
> In addition to these sentences in the results:
>
> *Delivering on the COP28 tripling pledge will require an estimated 8,000 GW of renewable capacity to be deployed. In generation terms, this translates to around 23,000 TWh of renewable generation[3] - mainly from onshore wind and solar PV - which is roughly 2.5% of the global technical potential calculated in **Error! Reference source not found.**.*
>
> *High costs of capital have implications on both the equity and pace of the energy transition[22], as well as the effectiveness. Meeting the COP28 tripling renewables target by deploying wind and solar in locations with high grid carbon intensities and higher capacity factors will deliver far larger renewable generation and emissions reductions per MW of installed capacity[46].*

EMDEs and China will need to play a larger role in achieving the COP28 tripling renewables target, though current ambitions may need to rise from 2.4 to 3.4 to meet rapidly rising electricity demand and displace fossil power from national grids. High costs of capital mean that the scales of abatement required in developing regions cannot be met at cost competitive LCOEs, for both solar and onshore wind.

In addition, as discussed below we have linked the calculation of total increases in cost in EMDEs to the COP28 target:

Delivering on the COP28 tripling pledge will require an estimated 8,000 GW of renewable capacity to be deployed. In generation terms, this translates to around 23,000 TWh of renewable generation[3] - mainly from onshore wind and solar PV - which is roughly 2.5% of the global technical potential calculated in ***Error! Reference source not found.****. Around 5 ,000 GW of the additional capacity will need to be concentrated in EMDEs, based on modelling conducted by the IEA[51]. High costs of capital in EMDEs lead to an annual increase of US$354 and US$181bn for onshore wind and solar PV (US$7.1 and 3.6 trillion across their lifetime), if the additional 5,000 GW was deployed in the best locations in EMDEs (see* ***Methods****).*

And the following into the discussion:

High costs of capital for renewable investments are a substantial barrier to the energy transition in developing countries, which could substantially reduce the overall mitigation potential of meeting the COP28 tripling renewables target by impeding deployment in locations with stronger abatement potentials. Addressing the barrier of high costs of capital through policy interventions and concessional finance will be essential to an equitable, rapid and effective energy transition[27].

Lowering the cost of capital to ensure that demand growth is met predominantly by clean energy will be essential to avoiding fossil lock-in, as well as ensuring that deployment of the additional 8,000 GW needed for the COP28 goal leads to both the maximum renewable electricity generation and kilotonnes of greenhouse gases abated.

However, the text tends to be overly descriptive and could be significantly improved in terms of structure and engagement, especially if the scenario analyses became more meaningful.

Response: We thank you for your feedback on how the manuscript could be improved. We have reviewed the manuscript throughout, adding and strengthening links to the COP28 tripling renewables goal and implications of high costs of capital to mitigated emissions.

As discussed below, we have also tried to improve the clarity and narrative of the text throughout, including through:

- We have cut down the introduction from 9 paragraphs to 4 paragraphs, making the purpose statement of the paper much earlier to ensure clarity and conciseness.

> - We have cut several sentences from the "High costs of capital hold back cost-competitive renewable potential" section, including the following:
> - We have conducted two full passes of the text and corrected pluralisation's, articles, duplicated and missing words to improve the textual clarity and quality.

Specific comments:

Lines 58–64, page 2: The distinction between points (1), (3), and (4) is unclear. Please clarify how these are conceptually different. How one is sure that 3 and 4 are not included in 1?

> **Response:** The basis for points (1) to (4) was to capture how the key variables influencing the levelised cost of electricity have changed over recent decades and the drivers behind them, split into falls in capital expenditures (1), discount rates (cost of capital, 2), operating costs (4) and [increases in] capacity factors (3)
>
> In response to your comment we have adjusted the language to the following to make it clear to readers that these are conceptually different and the underlying rationale for their selection:
>
> *Cost reductions have had four major drivers: falling capital costs due to experience and economies of scale[12]; decreasing cost of capital from financial learning[13] and periods of low interest rates[14]; technical innovations increasing the average capacity factor[15]; and lower operating and maintenance costs[16].*

Line 65: High-income countries also provide fossil fuel subsidies. Please revise to reflect this nuance.

> **Response:** Following a reworking of the Introduction to improve brevity – as suggested by Reviewer #1 - we have removed the reference to fossil fuel subsidies.

Lines 104–105: "Africa" is repeated twice—please correct.

> **Response:** Thank you for bringing this to our attention – we have removed the repetition.

Line 111: Please rephrase for clarity. "MW" refers to capacity, not to electricity or energy per se.

> **Response:** We have rephrased the passage to the following:
>
> *Bringing down high costs of capital in EMDEs and accelerating renewables deployment through de-risking efforts[44] will ensure the decarbonisation of the global energy system is more efficient both in terms of energy generation[45] (by deploying capacity in locations with high capacity factors) and mitigation potential[46] (by deploying capacity in locations with high grid intensities).*

Figure E: Panels are labeled “c” and “d” but should be “a” and “b”.

> **Response:** We are not sure which Figure you are referring to, and so have checked all those with labelling that includes c and d – we assume that this was in reference to Figure 3 as that is the only one with a “d” panel. Following comments from other reviewers, we have merged c and d to give on consolidated average LCOE increase, so the figure now reads absolute increase solar (a), wind (b) and then the average increase across both solar and wind (c).

Lines 222–223: The 5% increase is not clearly explained. Is this uniformly distributed? Please specify how this numbers were derived and applied.

> **Response:** In response to your comment and your earlier one about the links to the COP28, we have revised our calculation to be more strongly and clearly linked to the COP28 goal:
>
> *Delivering on the COP28 tripling pledge will require an estimated 8,000 GW of renewable capacity to be deployed. In generation terms, this translates to around 23,000 TWh of renewable generation[3] - mainly from onshore wind and solar PV - which is roughly 2.5% of the global technical potential calculated in **Error! Reference source not found.**. Around 5 ,000 GW of the additional capacity will need to be concentrated in EMDEs, based on modelling conducted by the IEA[51]. High costs of capital in EMDEs lead to an annual increase of US$354 and US$181bn for onshore wind and solar PV (US$7.1 and 3.6 trillion across their lifetime), if the additional 5,000 GW was deployed in the best locations in EMDEs (see **Methods**).*
>
> And we have included the basis of the calculation as a new subsection in the Methods:
>
> *Evaluating the potential increase in costs to meet the COP28 tripling target caused by high costs of capital in EMDEs is challenging, due to uncertainties around a) regional distribution of new capacity, b) density of deployed capacity and c) non-technical factors that influence deployment (e.g., grid capacity, conflicting land uses, distance from demand centres, etc). To give an upper bound for the extent to which high costs of capital in EMDEs increase the annual costs of meeting the target, the following steps are undertaken: (1) locations in developed countries are removed for each technology; (2) the lowest cost locations to meet the 5,000 GW target are extracted, based on an assumption of 1 MW deployed at each location (i.e. 5m locations selected); (3) the average cost increase per year is calculated, across each location, using Equation [1]; and (4) the average cost increase was summed across the 5m locations in EMDEs across the world.*

$$Average\ cost\ increase = \frac{\sum_t^n E_t * (LCOE_{national} - LCOE_{uniform})}{n} \qquad [1]$$

Scenario design: The comparison between uniform and national scenarios lacks a clear narrative. It is difficult to understand the logic or policy relevance behind the scenario choices and what message the authors intend to convey.

> **Response:** The two cost of capital "scenarios" were selected to indicate the marked difference that high costs under current perceptions of investment risk have on the levelised cost of electricity in EMDEs, by providing estimates of the LCOE under "National Scenario" costs of capital and the average cost of capital in developed countries (the "Uniform Scenario". Calculating and illustrating the delta as in Figures 3 and 5 thereby show the extent to which current economic and financial systems drive a disparity between locations with the highest renewable potential and the lowest levelised costs of electricity, which we then use as an analytical basis to discuss implications for renewable investment and emissions abated from renewable deployment.
>
> However, we recognise that labelling these as "Scenarios" is arguably a misnomer; as they do not represent and are not based on explorations of future changes to technology costs, policy environments or socioeconomic changes. In response to your comment, we have removed the term scenario from throughout the paper and replaced with "uniform costs of capital" or "nationally-specific costs of capital".
>
> We have also inserted the following sentences into the **Modelling the Cost and Scale of Renewable Potential** subsection to outline the logic and rationale for the selection of these two calculations:
>
> > *These two bases for calculation (referred to as National and Uniform) were selected to illustrate the extent to which high costs of capital in EMDEs push up the LCOE, impairing the economic viability of renewables and so leading to their deployment being largely concentrated in countries with lower grid carbon intensities.*

Figures: There appear to be two different "Figure 3"s. Please fix this inconsistency.

> **Response:** Thank you for spotting this. We have corrected all Figure numbers to ensure that there is no repetition.

Throughout the text: Some repetitions could be removed for clarity and conciseness. Consider tightening the writing to improve readability and flow.

> **Response:** Thank you for your comment. In response, we have conducted a fully detailed review, removing any areas which felt repetitive to improve clarity, readability and the overall flow of the manuscript.
>
> For example:
>
> We have cut down the introduction from 9 paragraphs to 4 paragraphs, making the purpose statement of the paper much earlier to ensure clarity and conciseness.
>
> We have cut several sentences from the "High costs of capital hold back cost-competitive renewable potential" section, including the following:

> *Countries currently in military, political or macroeconomic crises (e.g., Ukraine, Venezuela, South Sudan) see the largest increases in LCOE.*
>
> *Under national costs of capital, this falls to 295 and 86 TWh in each region (15 and 5% of regional offshore potential). The cost of capital is even more important to offshore wind project viability given the scale and capital intensity of projects, which explains why many offshore wind developers have paused projects due to recent rises in global interest rates*[58].

We have removed the following sentence from the "Global Renewable Energy Potential":

> *Reduced competitiveness of renewables from high costs of capital risks further lock-in of fossil fuel infrastructure across EMDEs*[7]*, with implications for both the equity and success of the global energy transition without international support*[22,23,50].

The Work offers a useful data resource, the analytical framing and textual clarity require further refinement to fully realize its potential contribution.

Response: We are glad to hear that you think the work offers a useful data resource; following the reviews that were received we have also uploaded the underlying data to a Zenodo repository (https://zenodo.org/records/17798380) to maximise the utility of the modelling that we have conducted to the wider research community.

In response to your comment here and above highlighting the need for further refinement, we have conducted two full passes of the text and corrected pluralisation's, articles, duplicated and missing words to improve the textual clarity and quality.

To improve the analytical framing, we have made a stronger link between the LCOE, CoC and lifetime abatement potentials and their implications for the global energy transition throughout the manuscript, including a full restructuring of the introduction which explicitly makes these links:

> *Marked differences in the geographic distribution of high quality renewable energy resources and accessible costs of capital for project developers are a key driver of the concentration of deployment, often out of line with locations that have the best renewable potential*[43]*. Whilst this holds implications for cost and equity of the global energy transition*[22]*, the implications on the overall effectiveness of mitigation efforts and progress towards the COP28 goal have not yet been explored in depth. As EMDEs often have higher current grid intensities and/or larger planned project pipelines of fossil power capacity, deploying renewables would deliver much larger mitigation per unit of capacity. Bringing down high costs of capital in EMDEs and accelerating renewables deployment through de-risking efforts*[44] *will ensure the decarbonisation of the global energy system is more efficient both in terms of energy generation*[45] *(by deploying capacity in locations with high capacity factors) and mitigation potential*[46] *(by deploying capacity in locations with high grid intensities). However, there has been limited examination of the interplays between renewable LCOEs, costs of capital and lifetime abatement potential of renewables in the literature. Here, using a global geospatial model, we present global estimates of the levelised cost of electricity (LCOE), annual technical potential and lifetime abatement of solar, onshore wind and offshore wind power at a high*

> *geospatial granularity, using country- and technology-specific costs of capital for over 150 countries in 2024. Combining the technical and economic analysis, we evaluate how much cost of capital raises the LCOE across over 96,000 grid points, the impact that this has on regional cost-supply curves and implications for national and regional progress towards the COP28 goal. We find that locations in Africa, Central Asia and South America would have levelised costs within or below US LCOE ranges, if countries could access developed countries' financing terms, with the share of financing costs in the overall LCOE rising from 40% to nearly 76%, an average increase in EMDEs of US$21/MWh (solar) and US$26/MWh (onshore wind).*
>
> In the results we have also made the link between high costs of capital and abatement more strong, as well as the link to the COP28 goal:
>
> *High costs of capital have implications on both the equity and pace of the energy transition[22], as well as the effectiveness. Meeting the COP28 tripling renewables target by deploying wind and solar in locations with high grid carbon intensities and higher capacity factors will deliver far larger renewable generation and emissions reductions per MW of installed capacity[46].*
>
> *EMDEs and China will need to play a larger role in achieving the COP28 tripling renewables target[51,61], though capacity may need to more than triple to both displace fossil power from national grids and meet rapidly rising electricity demand. High costs of capital mean that the scales of abatement required in developing regions largely cannot be met at cost competitive LCOEs, for both solar and onshore wind.*
>
> In the discussion, we have also highlighted these links throughout to strengthen the analytical framing:
>
> *High costs of capital for renewable investments are a substantial barrier to the energy transition in developing countries, which could substantially reduce the overall mitigation potential of meeting the COP28 tripling renewables target by impeding deployment in locations with stronger abatement potentials.*

Reviewer #2 (Remarks to the Authors (required))

The paper estimates the impact that national cost of capital values have on LCOE and subsequently on the carbon abatement potential. It argues that developing countries have higher costs of capital, which in turn increases the LCOE in comparison to the uniform cost of capital (CoC) scenario. As developing countries have the highest CoC and among the highest renewable energy potential (especially solar), a lot of renewables capacity additions are being held back. The high CoC hinders project development in those countries, compared to developed and industrialized countries (like China).

The paper is of high quality, and it is very well written and structured. The images are compelling and easy to understand, with some exceptions (see below). The paper also cites all the relevant literature. Nevertheless, I still have some comments for the authors:

> **Response:** We thank you for your engagement with the paper and for your valuable comments. We are glad to hear that you think it is of high quality, well written and effectively structures. We have responded to all of the points that you raise and made revisions to the manuscripts in line with your recommendations accordingly to 7 out of 8 of your comments, and for the other case have included a detailed response on why we feel a revision is not necessary to address your concerns.

Major comments

The first main result (starting in line 205) is already known to the field. Differences in costs of capital obscure LCOE levels, making them lower in developed and higher in developing countries. Hence, this is not such a major novelty. I wonder if this is really needed in the main body of the text. If the aim is to present this paper to wide audiences that are not familiar with the subject, then yes. If the target group are experts in the field, than no. In addition, Figure 1 seems to repeat itself three times.

> **Response:** We thank you for your comment and for highlighting that this aspect of the results section is already well known and so may not be needed in the text. Whilst differences in cost of capital and how these feed through to the LCOE of renewable technologies has already been well discussed in the field, to date existing cost of capital estimates have not been applied within a geospatial LCOE model at the levels of spatial and financial granularity that we present here. We aim in our manuscript here to present the paper and results both to experts in the field and Nature Communication's wider audience who may not have in-depth knowledge of the field of energy economics. Given these two factors and in line with your comment, we have kept the results in the main body of the text.
>
> We also thank you for bringing to our attention that Figure 3 (we believe this is the figure you are referring to as Figure 1 is the modelling schematic) appears to repeat itself three times, in Fig3a, 3c and 3d. Whilst we believe it is important to include both the percentage and absolute increases in LCOE for solar and wind, Figures 3c and 3d have very similar percentage increases due to relatively small differences in the cost of capital for wind and solar (under the minor influence of technology risk premiums compared to other parameters). We have merged these into a new Fig3c which reflects the average increase in LCOE across both wind and solar (onshore) and for wind (offshore) and adjusted the caption accordingly.

2. Connected to this, the main novelty I see in Figure 1 is that the LCOE are calculated on a very granular level. This is an interesting methodological contribution; however, I wonder why is it needed? The macroeconomic risk which drives the CoC is the same for the entire

country. Hence why show LCOE in higher resolution? I would ask the authors to better explain the relevance of this granular approach. Figure 1 seems to be different mainly along the country lines anyway. Probably there are LCOE differences within the country, because of the different solar and wind potential distribution, but this is not seen from the map because of the global focus.

> **Response:** We thank you for your recognition of the novelty outlined in Figure 1 (3) with the calculation of the LCOE at a very granular level. Our paper combines country-level estimates of the cost of capital with regional and country-specific deployment costs and a geospatial model with high spatial granularity, which to date has not been performed. This approach allows for sub-national differences in wind and solar potential to be accounted for, as highlighted, and for the calculation of potentials in Figures 2 and 5 to be conducted.
>
> In response to your comment, we have added the following lines into the "Modelling the Cost and Scale of Renewable Potential" section to more clearly underline the relevance and justification for calculating the LCOE at such a high resolution:
>
> *First, we develop hourly power output profiles of solar PV[47] and wind power[48] globally at a high geospatial granularity to accurately depict sub-national distributions of renewable potential.*
>
> *Finally, the renewable abatement potential at each grid cell location is also calculated using technical potential, renewable lifetimes and grid carbon intensity scenarios; which is used to generate LCOE-abatement curves based on the highly granular geospatial approach.*

In my view the main contribution of the paper is that high CoC slow down the uptake of renewables in countries where the abatement potential is the highest. I did not yet see someone making this connection. However, this result is presented only later in the results section, although I believe it's the most relevant one. I would urge the authors to give this result more prominence in the text. The authors do mention the abatement potentials in the intro. However, it took me until the results section to finally understand why the authors are calculating the abatement potentials. It needs to be better explained in the intro. The connection between CoC, LCOE and abatement potential is not clear.

> **Response:** We thank you for highlighting the main contribution of the paper, a connection which as you rightly note has not been made to date and that we believe is the key and most relevant result of our paper.
>
> In response to your comment, we have reformulated the abstract to explicitly make this connection:
>
> *Using grid carbon intensity trajectories, we show that the largest carbon abatement potentials are in developing countries, over 20 times those in developed countries, with high costs of capital a key barrier to deployment in many locations (excluding China).*

> We have also included a paragraph in the introduction that explicitly highlights the links between CoC, LCOE and abatement, following a review and restructuring of the introduction which also makes this link more clearly both throughout and earlier:
>
> > *Marked differences in the geographic distribution of high quality renewable energy resources and accessible costs of capital for project developers are a key driver of the concentration of deployment, often out of line with locations that have the best renewable potential[43]. Whilst this holds implications for cost and equity of the global energy transition[22], the implications on the overall effectiveness of mitigation efforts and progress towards the COP28 goal have not yet been explored in depth. As EMDEs often have higher current grid intensities and/or larger planned project pipelines of fossil power capacity, deploying renewables would deliver much larger mitigation per unit of capacity. Bringing down high costs of capital in EMDEs and accelerating renewables deployment through de-risking efforts[44] will ensure the decarbonisation of the global energy system is more efficient both in terms of energy generation[45] (by deploying capacity in locations with high capacity factors) and mitigation potential[46] (by deploying capacity in locations with high grid intensities). However, there has been limited examination of the interplays between renewable LCOEs, costs of capital and lifetime abatement potential of renewables in the literature. Here, using a global geospatial model, we present global estimates of the levelised cost of electricity (LCOE), annual technical potential and lifetime abatement of solar, onshore wind and offshore wind power at a high geospatial granularity, using country- and technology-specific costs of capital for over 150 countries in 2024. Combining the technical and economic analysis, we evaluate how much cost of capital raises the LCOE across over 96,000 grid points, the impact that this has on regional cost-supply curves and implications for national and regional progress towards the COP28 goal. We find that locations in Africa, Central Asia and South America would have levelised costs within or below US LCOE ranges, if countries could access developed countries' financing terms, with the share of financing costs in the overall LCOE rising from 40% to nearly 76%, an average increase in EMDEs of US$21/MWh (solar) and US$26/MWh (onshore wind).*

4. The authors suggest in the discussion that the costs of capital have to come down in developing countries. Since the CoC are mainly driven by country risk, how do the authors suggest to achieve this? The current suggestions sound a bit superficial. Concessional finance enables some projects to reduce the CoC. Its mainly arranged for projects that open a market – for instance, the first onshore wind farm in the country etc. Apart from this, there are good examples where multilateral finance institutions helped drive down risk. For instance, the World Bank in Serbia and its impact on making a bankable PPA, which has driven the domestic wind energy market. But this finance source / assistance will not be available to most of the renewable energy potential that needs to be developed. Small scale solar PV would have to rely on commercial banks. How should the small investors in Bangladesh tap into the concessional finance grants etc. and assistance? Are there some loan programs for microloans for solar PV? I would

ask the authors to be more specific about measures to reduce the cost of capital, perhaps by giving some more specific examples.

> **Response:** Thank you for your reflections on the discussion section of our manuscript; you are right to highlight that the high costs of capital in EMDEs are largely driven by country risk which current interventions (de-risking measures and concessional finance) may struggle to address at scale. In response, we have included the following paragraph which discusses the role of reforms to current credit rating agencies and scaling of domestic capital markets and how they will be key to addressing high costs of capital by bringing down country risk premiums:
>
> *Estimates show that annual clean energy investment into EMDEs will need to increase by more than six times by 2030 for the world to be on track for net zero by 2050[27]. Addressing high costs of capital will be essential to scaling private investment flows into EMDEs, which from our estimation here are largely held back by high country risk premiums. These conclusions align with findings from the IEA that an estimated 70% of the risk factors contributing to high costs of capital in EMDEs are caused by macroeconomic and currency risks[27] included in country risk premiums. These are informed by sovereign credit ratings and the "sovereign ceiling" effect[69], which an increasing body of literature has shown to be subjective[70,71] and structurally biased against EMDEs[72], underlining the need for reform of the structure and methodologies of current credit rating agencies. Data on default rates and returns from infrastructure and energy projects in EMDEs also does not reflect levels implied by the ceiling of sovereign credit ratings[73], demonstrating that it is largely the perception of higher risks that is dissuading investors. Improved project-level data availability will be essential to informing more risk-reflective behaviour from international investors, alongside scaling domestic capital markets and improving the enabling environment for investment. Successful initiatives for the former have included the development of micro-finance institutions and other forms of innovative financing for renewables. For example, local currency loans and the "pay-as-you-go" model provided by Sun King have led to over 27m of solar products distributed across Africa (with support from concessional loans delivered by the IFC)[74].*
>
> This paragraph also includes explicit discussion of how scaling domestic capital markets can support small-scale wind and solar and the role that microloan programmes can play. We have also included specific examples of successful de-risking efforts from multilateral financial institutions into the preceding paragraph (on concessional financing and de-risking efforts):
>
> *Reform of the global financial architecture to better deliver investment at affordable rates into EMDEs faces many economic and geopolitical barriers. In the short term, support from international public financiers (IPFs) will be essential to effectively de-risking projects and "crowding in" the private investment required, both by serving as an anchor point and addressing underlying policy risk factors[75]. Support can include loan guarantee programmes[76], strengthening national regulatory frameworks[27] and a combination of capacity building and technical assistance[77]. Examples of successful interventions have*

included risk guarantees and political insurance for Ugandan hydroelectric power[78], support with the development of bankable PPAs in Serbia[79] and efforts to support enabling environments for private investment into clean energy by the World Bank[80]. IPFs can also provide finance at concessional rates[23], which is critical for the least developed countries that we show here face some of the highest commercial costs of capital. Estimates show that concessional energy-related funding for clean energy investment will similarly need to scale up by five to six times to ensure that clean energy investment in developing countries reaches a level that delivers on the Paris Agreement[81,82]. The New Collective Quantified Goal for climate finance, agreed at COP29[83], included an ambition to expand international public and concessional financing but was criticised for lacking sufficient inclusion of grants and other below-market sources of financing[84]. Here, we show that Sub-Saharan Africa, Latin America and parts of Asia see high costs of capital that severely limit renewables feasibility and their deployment in locations with high abatement potentials. International support in these regions would therefore be most impactful to accelerating effective progress towards the COP28 goal and maximising the emissions reductions enabled by the 8,000 GW of capacity required to achieve the target.

Minor comments

In Figure 5 and Figure 7 it would be useful to have a list of abbreviations for the regions. Perhaps a table in the Appendix listing the countries within one region.

Response: We agree that it would improve readability to have a list of abbreviations for the regions. We have added into the Supplementary Data a list of the countries within one region (in addition to a citation to the TIMES region groupings) and as well as a list of abbreviations for the regions in Figures 5 and 7 as follows:

AFR: Africa, AUS: Australia and Oceania, FSU: Former Soviet Union, CAN: Canada, CHN: China, CSA: Central and South America, IND: India, JPN: Japan, MEA: Middle East, MEX: Mexico, ODA: Other Developing Asia, EEU: Eastern Europe, KOR: Korea, USA: United States of America, WEU: Western Europe

The authors use post tax WACC. The WACC rates here depend also on the country tax rates. Hence, the final WACC values used could be affected by the country's taxation rules. I think that post tax WACC can still be used as the authors are probably looking at the private economic perspective. However, I think it would be useful to add this as a note in the text.

Response: Thank you for bringing this to our attention, as you highlight the final WACC values are affected by the country's taxation rules which we include in our estimates of the cost of capital based on Equation 2. However, due to an oversight in the previous draft we had not included the data source used for country-level taxation rates in the Methods section. We have now included the following sentence into the Methods:

Tax rates (τ^x) at a national level were extracted from reporting on corporate tax rates from the Tax Foundation[101]. Whilst many countries include a range of industry-specific tax

> *incentives that may affect the overall cost of capital for renewables, due to the lack of data comparing these interventions across countries the tax rate was assumed to be the same as the standard corporate tax rate in each jurisdiction. We note that renewable-specific tax rates may lead to our estimated costs of capital being higher based on these limitations, given that some countries provide preferential taxation schemes for clean energy projects to encourage investment.*

3. The debt ratio values are now in a range that is informed by the Diacore project. I would like to highlight to the authors that there were newer studies like Diacore, such as the AURES2 cost of capital survey for Europe.

> **Response:** Thank you for bringing this to our attention – we have compared the debt ratio values based on Diacore to AURES2 and found these to still be in line with the inverse linear relationship used to relate country risk premium and debt ratios, with reported values from AURES2 ranging down to lows of 40% (exc. fully on-balance sheet financing in Spain and Portugal). We have adjusted the sentence in the methods to the following to reflect that data from both the AURES2 and Diacore projects were used:
>
> *The boundaries of 40-80% were informed by the reported ranges in the Diacore and AURES2 projects for the EU-27, which ranged between 40-80% for solar PV and onshore wind[102,103] (excluding results from Portugal and Spain, where some projects were financed with 100% equity through corporate financing structures).*

4. I would restructure the into so that the main purpose statement of the paper comes much sooner. Now it comes in paragraph 9. It should come the latest until paragraph 4. I would urge the authors to come to the point much sooner.

> **Response:** We thank you for your feedback on the paper introduction. We acknowledge that the purpose statement of the paper is relatively late in the introduction, with a number of paragraphs that include background context and key information that justifies the scope and purpose of the paper. We have fully reviewed the introduction and shortened throughout, merging paragraphs where possible and including more explicit references to the limitations of existing data and research; the main purpose statement now comes at the end of paragraph 4. The new final paragraph reads as following:
>
> *Marked differences in the geographic distribution of high quality renewable energy resources and accessible costs of capital for project developers are a key driver of the concentration of deployment, often out of line with locations that have the best renewable potential[43]. Whilst this holds implications for cost and equity of the global energy transition[22], the implications on the overall effectiveness of mitigation efforts and progress towards the COP28 goal have not yet been explored in depth. As EMDEs often have higher current grid intensities and/or larger planned project pipelines of fossil power capacity, deploying renewables would deliver much larger mitigation per unit of capacity. Bringing down high costs of capital in EMDEs and accelerating renewables deployment through de-risking efforts[44] will ensure the decarbonisation of the global energy system is more efficient both in terms of energy*

> *generation[45] (by deploying capacity in locations with high capacity factors) and mitigation potential[46] (by deploying capacity in locations with high grid intensities). However, there has been limited examination of the interplays between renewable LCOEs, costs of capital and lifetime abatement potential of renewables in the literature. Here, using a global geospatial model, we present global estimates of the levelised cost of electricity (LCOE), annual technical potential and lifetime abatement of solar, onshore wind and offshore wind power at a high geospatial granularity, using country- and technology-specific costs of capital for over 150 countries in 2024. Combining the technical and economic analysis, we evaluate how much cost of capital raises the LCOE across over 96,000 grid points, the impact that this has on regional cost-supply curves and implications for national and regional progress towards the COP28 goal. We find that locations in Africa, Central Asia and South America would have levelised costs within or below US LCOE ranges, if countries could access developed countries' financing terms, with the share of financing costs in the overall LCOE rising from 40% to nearly 76%, an average increase in EMDEs of US$21/MWh (solar) and US$26/MWh (onshore wind).*
>
> As above, we have also included a stronger focus on the links between LCOE and mitigation/abatement potential with an explicit discussion of the fact that renewables are largely not being deployed in countries with high abatement potentials to better underscore the results and discussion sections of the manuscript.

Reviewer #3

The paper makes an important contribution in terms of raising the issue of high cost of capital in developing countries as an important barrier to upscaled implementation of RE and supporting the argument with empirical evidence.

> **Response:** We thank you for their reflections on our paper and recognition of the issue of high costs of capital in developing countries. We have responded to each of your remarks and revised the manuscript in response to them.

2. While there are data gaps for some country/region level data points, the overall point is well supported and availability of the code would enable researchers to replicate and test results in future.

> **Response:** We are glad that you think the data and results support the overall point well with availability of the code enabling researchers to replicate and explore results further in future. We have also included the underlying results in a Zenodo repository (https://zenodo.org/records/17798380) along with details of the cost of capital and LCOE under the two cost of capital assumptions at each location.

We have also developed and implemented a methodology to address the remaining country/region data gaps and have included the following text outline the approach into the methods:

For countries without a reported country risk premium, their country risk was set to the highest reported value in their region given that these countries are regarded as un-investable (i.e. "non-investment grade speculative") on international capital markets due to extremely high country risk factors[56]. and updated all results and figures throughout based on these changes.

3. Discussion section: Lines 436-437: The point about using accurate, context specific data to enhance usefulness of energy modeling, especially in developing countries could be further strengthened by extending the use of such data also in global Integrated Assessment Models (IAMs) that seek to examine alternative scenarios for burden sharing and decarbonization across regions and countries while aligning with global cumulative GHG emission limits.

Response: We thank you for your suggestion. This is something we are currently working on, so we are glad to see you highlighting this area. In response to your feedback, we have included the following passage into the text:

The use of context-specific data will also be valuable to global Integrated Assessment Models to explore alternative scenarios for burden sharing and decarbonization that account for equity implications, which will be an increasing focus under the IPCC's AR7 review[95].

Message to all Reviewers

We thank the reviewers for their positive assessment and constructive comments that have helped us to improve this manuscript. We respond to each point below in blue and new text added to the manuscript is quoted *in italics*.

We have responded to the remaining reviewer comments,

In addition to addressing the reviewer comments - clarifying the role of sovereign credit ratings in influencing project-level costs of capital (CoC) and more strongly emphasising the novelty of our paper in linking CoC, renewable potential and grid carbon intensity - we have edited the manuscript throughout to:

- We have updated the inputs to our LCOE model, previously for 2024, re-running it with CoC estimates for 2026 and renewable deployment costs (regional and national) published by IRENA in 2025 to present the most up to date picture of renewable project economics in the draft manuscript, updating all figures, numbers in the text and supplementary data accordingly.
- We have included an additional figure (Figure 7) that demonstrates the links between CoC, renewable resources and mitigation potential, as highlighted below, including data on the future project pipeline of generation projects to highlight how EMDEs are more likely to have more fossil power planned (with discussion of the role of CoC in affecting these findings).
- We have converted all references to "abatement potential" to "mitigation potential", to more closely align with other literature on this topic.
- We have conducted a thorough review and tightened up all language throughout, to maximise readability.

Reviewer #1

I think the authors have responded in a satisfactory way to the comments made.

> **Response:** We thank you for your valuable feedback and comments on our manuscript draft, we are glad that you are happy with our responses to the comments that have been raised.

Reviewer #2 (Remarks to the Authors (required))

1.Thank you for your revisions! This will be a fine paper. Still, I want to challenge you on one main account, and this relates to the novelty of your work. I can see that combining cost of capital, LCOE and grid intensities is novel as it enables us to see how crucial financial is for pushing renewables where they matter the most. Still, in your text you seem to put an equal amount of emphasis on the findings that LCOE is higher in developing countries when using non-uniform CoC etc. In my view this is not novel and it has been covered in the literature many times, as you also rightly indicate in the intro. This for instance relates to the finding from the abstract: "We show that high costs of capital 16 substantially increase LCOEs in developing countries, adding an average of US$21 (solar) and 17 US$26 (onshore wind). Financing costs constitute up to 78% of the LCOE, compared to 40–42% under uniform global costs of capital".

You write: "Combining the technical and economic analysis, we evaluate how much cost of capital raises the LCOE across over 96,000 grid points, the impact that this has on regional cost-supply curves and implications for national and regional progress towards the COP28 goal. We find that locations in Africa, Central Asia and South America would have levelised costs within or below US LCOE ranges, if countries could access developed countries' financing terms, with the share of financing costs in the overall LCOE rising from 40% to nearly 76%, an average increase in EMDEs of US$21/MWh (solar) and US$26/MWh (onshore wind)."

If you are Sudan and have points A and B on your grid, the difference between A and B in costs of capital will be the same. Then the LCOE difference comes from renewable energy potential and not cost of capital. Hence, I would again like to question if you should really stress this as the most important aspect of your paper. In summary: grid carbon intensity plus bigger potential in developing countries and the lack of using this if financing is not there, is in my view the main hook of the paper, whereas "look we model 96000 points" and show differences between developed and developing countries is not that novel in my view.

Response: We agree that the existence of higher LCOE in EMDEs due to higher costs of capital is well established, and we do not intend to position this as the primary novelty of the paper. We have therefore reframed the manuscript so that the central contribution is the interaction between (i) high grid carbon intensity, (ii) high-quality renewable potential, and (iii) high costs of capital for renewable projects in EMDE, and how this combination suppresses the greatest opportunities for effective mitigation. Concretely, we revised the abstract and the end of the Introduction to lead with this interaction and treat average LCOE uplifts as supporting context (see revised text quoted below, with line references in the manuscript).

- We have completely restructured the abstract, which now reads: *"High costs of capital are a substantial barrier to renewables deployment in emerging markets and developing economies (EMDEs), impeding energy system decarbonisation and risking the lock-in of fossil power generation even in countries with strong renewable potential. Using grid carbon intensity trajectories, we show that the largest mitigation potentials per project are in EMDEs, over 20 times those in developed countries even before accounting for planned fossil power projects. However, high costs of capital substantially increase the levelised cost of electricity (LCOE) in EMDEs, adding an average of US$26/MWh (solar) and US$24/MWh (onshore wind) compared to if they could access developed country financing terms. Financing costs account for up to 78% of renewable LCOEs in EMDEs, compared to 37-39% in developed countries, restricting a large portion of global technical potential. We estimate that this could lead to an annual cost increase of US$245bn and US$329bn for solar and wind respectively under the UNFCCC target of tripling renewables by 2030, or US$5.0–6.6 trillion across projects lifetimes. Our results emphasise the importance of reducing the cost of capital in EMDEs for an equitable and effective transition, through accelerating renewable deployment in locations with strong potential and larger lifetime mitigation of emissions."*
- At the end of the introduction (lines 98-103), we have edited the final two sentences to read: "*Combining the technical and economic analysis with grid carbon intensity trajectories, we find that deploying wind and solar in EMDEs could deliver over twenty times the lifetime mitigation per renewable project than in developed countries, but that high costs of capital substantially increases renewable LCOE in EMDEs, by an average of US$26 (solar) and US$24 (onshore wind) and slowing more effective progress towards achieving the UNFCCC renewables target of tripling through harnessing high-quality renewable potential in EMDEs*"
- We have also included the following paragraph into the "Renewable mitigation potential" section (lines 326 – 350), with a corresponding figure, to more strongly emphasize the link between CoC, renewable resources and

mitigation potential, as well as include discussion of future deployment of fossil power being concentrated in EMDEs: "*Excluding Australia and Poland, which still have substantial coal power on their grids[62], it is predominantly developing and industrialising countries that have the highest grid intensities (see **Extended Data Figure 6**) and so see the highest lifetime mitigation potential from renewable deployment. We illustrate in Error! Reference source not found. that it is almost exclusive EMDEs where the project pipeline of fossil power exceeded renewables by mapping the cost of capital, mitigation potential and ratio of planned fossil to renewable power by country, with solar and wind deployment in many EMDEs able to deliver much higher mitigation potentials per than median levels in Annex II countries. Accounting for the future pipeline of planned fossil power additions[7] - which is largely focused in EMDEs - would make deploying renewables in EMDEs even more effective in mitigation terms. Many EMDEs with strong renewable potential (measured through median capacity factor) that face high costs of capital for renewables have a much larger pipeline of fossil power generation projects than renewable projects in MW terms, illustrating that high costs of capital could be one of the factors holding back the deployment of renewables to meet energy demand growth in EMDEs. Without interventions to reduce the cost of capital and accelerate deployment, the planned fossil project pipeline in EMDEs could lead to carbon lock-in and risk missing global emissions targets under the Paris Agreement. In some EMDE regions, limited renewable deployment despite ample potential has already led to fossil power deployment. For example, despite having some of the strongest solar resources globally, the continent of Africa has seen an increase of 52% in gas generation over the last decade[63] with nearly 60 GW of gas power currently in development to meet future rises in demand from economic and population growth[61]. Solar and wind could provide an alternative power generation source to meet rising electricity demand, which could deliver substantial mitigation potential if displacing planned fossil generation capacity, but as we show here their competitiveness is held back under high costs of capital in EMDEs which push up their LCOEs.*

- We have also included the following sentence into the start of the discussion to emphasis the interactions between high CoCs and abatement as the central findings and technical insights from our paper (lines 389 – 397): "*Here, through a global technoeconomic evaluation of the technical potential, levelised cost and mitigation effects of solar, onshore and offshore wind generation, we show that high costs of capital are holding back the harnessing of high-quality renewable potential in emerging market and developing economies (EMDEs), by causing an average increase in LCOE of US$26, 24 and 40/MWh (for solar PV, onshore and offshore wind, respectively). Given that EMDEs typically have much higher grid carbon intensities and pipelines of*

> *planned fossil projects, we show that the lifetime mitigation potential from deploying wind or solar power could exceed 20x that in advanced economies, but that high LCOEs in those locations are a barrier to deployment."*

Connected to this, could you show if there are any intra country variations in your LCOE values that are driven by factors other than country risk. Seeing this variability would justify your granular approach, although, as stated earlier I would be surprised if you had large variations that are driven by CoC within a single country.

> **Response:** Thank you for your comment, we agree that this would be a useful addition. As we only model deployment costs and the CoC at a national level, intra-country variations in LCOE values are driven by the quality of the renewable potential at each location (i.e., the yearly average capacity factor). In response, we have taken the following actions:
>
> - Included new figures (ED Figures 7 and 8) demonstrating how LCOE varies with capacity factor within countries and regions modelled in the study, for both solar and wind, with the estimated national cost of capital illustrated through shading.
> - Included the following sentence into the "High costs of capital hold back cost-competitive renewable potential" section (lines 180 – 183): *Differences in the changes in the LCOE within countries are influenced by the distribution of renewable potential, with sites with poor renewable potential seeing a larger increase in the LCOE under national costs of capital (as it increases the LCOE proportionally to the level under the uniform calculation).*"
> - Included the following sentences into the "Global Renewable Energy Technical Potential" section (lines 134 – 140), which now reads: "***Figure 2*** *presents the annual solar, onshore wind and offshore wind technical potentials globally, obtained by applying our model with minimum capacity factor cut offs (see* ***Methods****) across over 96,000 locations. Using a geospatial LCOE model with such a high spatial granularity allows us to explore more effectively the effects of a) intra-country variations in renewable resource (i.e., capacity factor), b) land use and availability constraints that reduce the technical potential and c) explore the lifetime abatement potential at each location at a higher granularity.*
> - Included the following sentences into the final sentence of the Discussion to highlight that intra-country variations in the cost of capital are a key area for future study (lines 511 – 516) "*Further efforts should be focused…. accounting for subnational differences in the cost of capital for renewable firms*[13] *and*

> *projects[96,97] caused by project- and company-level risk factors not modelled here."*

2.In the new paragraph in the discussion section (918 – 984 in the Track Changes version) you now discuss a very specific example, which I like and this seems to address my comment directly. In there you write: "These are informed by sovereign credit ratings and the "sovereign ceiling" effect69, which an increasing body of literature has shown to be subjective70,71 and structurally biased against EMDEs72, underlining the need for reform of the structure and methodologies of current credit rating agencies. Data on default rates and returns from infrastructure and energy projects in EMDEs also does not reflect levels implied by the ceiling of sovereign credit ratings73, demonstrating that it is largely the perception of higher risks that is dissuading investors. Improved project-level data availability will be essential to informing more risk-reflective".
After reading this for the first time, I could not understand the term sovereign ceiling effect and how this relates to subjective risk perception. I would appreciate it if you could clarify that link.

> **Response:** We are glad to hear that you like the additional paragraph added to the discussion section. We have edited those sentences to define the sovereign ceiling effect and include further discussion on the links between sovereign credit ratings and renewable energy project costs of capital (lines 436 – 453) which now reads:
>
> *"These factors are largely accounted for in country risk premiums, which are derived from the sovereign credit ratings of a given country and are one of the largest drivers of high costs of capital in EMDEs. As corporate and project credit ratings are largely capped at the country's sovereign credit rating (a "sovereign ceiling" for private credit ratings which cannot typically be exceeded[72]), inaccuracies or misperceptions in sovereign risk can severely constrain project financing conditions. An increasing body of literature has demonstrated the subjectivity and distance bias in sovereign credit ratings for developing countries[73,74], as well as structural biases in their methodology against EMDEs[75], underlining the need for reform of the structure, methodologies and role of credit rating agencies to help reduce the cost of capital. For example, modular sovereign credit ratings could aid a more risk-informed approach by investors than attempting to reflect a wide range of risk factors into one score (some of which will be applicable to the economic performance of projects or corporates operating under a given sovereign)[76]. Data on default rates and returns from infrastructure and energy projects in EMDEs also do not reflect the risks of failure implied by the ceiling of sovereign credit ratings[77], demonstrating that it is largely a perception of higher risks that is dissuading investors. Improved project-level data availability will be essential to informing more risk-reflective behaviour from international investors, alongside*

> *scaling domestic capital markets and improving the enabling environment for investment."*

Message to all Reviewers

We thank the reviewers for their positive assessment and constructive comments that have helped us to improve this manuscript. We respond to each point below in blue and new text added to the manuscript is quoted *in italics*.

We have responded to the remaining reviewer comments,

In addition to addressing the remaining reviewer comments – improving the readability, clarifying the role of modular credit ratings and making the links more strongly between country level factors and high costs of capital throughout the paper, we have also edited the manuscript to:

- Conduct a detailed review of the language of the manuscript, editing where necessary to improve readability.
- Updated the labels in Figures 2, 5, 7 and 8 to include more information on the regions (2, 5, 8) and changing the labelling to ISO codes rather than country names (in Figure 7).
- For Figure 7, we have included bolded text to illustrate where the Annex II countries fall on the distribution - as well as switching the Y axis to the absolute mitigation potential per MW of deployed capacity in each country – to depict the dynamics more accurately between the CoC, mitigation potential and domestic renewable resources.

Reviewer #2

Thanks for the revision. I have only few smaller comments

> **Response:** We thank you for your valuable feedback and continued engagement with our manuscript draft.

1) To improve the readability of the introduction please separate the last paragraph into two, at the point where you write your purpose statement (line 94, page 3)

> **Response:** Thank you for the suggestion – we have separated the last paragraph of the introduction into two at line 94 (now 99), as well as also splitting the fourth discussion paragraph (now from lines 460 onwards) into two to improve readability.

2) In the discussion section, you write: "Failure to bring down the cost of capital in EMDEs will mean that less developed countries, including Bangladesh, will be unable to access the economic benefits of falling renewable deployment costs" In this sentence, and similar once before, you are saying that the high costs of capital is causing a higher LCOE and this is stopping investments. However, the high CoC is the consequence of overall country instability. For instance, the issue that there is widespread corruption, and as a developed you might need to heavily rely on connections to push your project or will need to engage in corrupt activities to get your permits higher. I suggest that you incorporate this connection with the higher CoC more - investors are not investing because of higher CoC caused by the lack of basic economic functions like rule of law etc.

> **Response:** We have rewritten the fourth paragraph of the discussion (now from lines 438 onwards) to incorporate the connection of country risk premiums and the higher cost of capital more strongly:
>
> *Estimates show that annual clean energy investment into EMDEs will need to increase by more than six times by 2030 for the world to be on track for net zero by 2050[27]. Targeted efforts to bring down the cost of capital in EMDEs will be essential to scaling private investment flows, which our modelling shows are largely held back by high country risk premiums. These conclusions align with the assessment by the IEA that an estimated 70% of the risk factors contributing to high costs of capital in EMDEs are caused by macroeconomic and currency risks[27], accounted for here in the country risk premiums. Factors influencing the country risk premium also include corruption, political structures, high exposure to violence and the strength of legal systems, challenges that pose difficulties in attracting investments beyond just the energy sector. Developing a strong enabling environment for investment will be important to scaling private investment to levels required to meet the UN Sustainable Development Goals, including through strengthening domestic institutions, reforming legal systems and designing regulatory environments to promote transparency and fair competition. Existing programmes to support domestic policymakers in addressing these issues include the UNDP's Governance for Anti-Corruption for Peaceful and Inclusive Societies Program and the World Bank's Improving Business Environment for Prosperity Program. For investors, political risk insurance (e.g., through the World Bank's MIGA program) can also provide coverage against country-level risk factors, including expropriation and breaches of contract, whilst for project developers, a dedicated investment facilitation unit for renewable project permitting could substantially reduce developers' exposure to corruption by streamlining approval pathways.*

We have also included references to the connections between country-level factors and broader challenges to investments in EMDEs throughout the manuscript, including in the following locations:

- In the second paragraph of the introduction (lines 64 – 66): *These risk factors also drive the high premiums that EMDEs pay for both public and private borrowing in sectors beyond clean energy, which cause difficulties in attracting external investment at affordable rates across sectors and is one of the drivers of the widening "financing gap".*
- In the "High costs of capital hold back cost-competitive renewable potential" section (lines 214-17): *Strengthening institutions, addressing corruption and pursuing increased political stability - although not directly targeting infrastructure investments - can play a key role in improving the investment landscape by reducing the country risk premium and so the cost of capital for clean energy investments.*

We have also separated out the points related to sovereign credit ratings into a separate paragraph (line 460 – 474) to improve readability and now discuss the link with country-level factors and high CoCs for energy projects directly in this paragraph.

Country risk premiums are largely derived from the host country's sovereign credit rating, as investors and businesses typically do not have the ability to conduct their own detailed macroeconomic and political assessments across countries of interest. Sovereign credit ratings act as a hard "sovereign ceiling" where projects or corporations are typically unable to be rated higher than the country rating[73], meaning that they are all but guaranteed a higher cost of capital than the sovereign borrowing costs. Any biases, inaccuracies and/or misperceptions of sovereign risk can therefore severely constrain project financing conditions under the "sovereign ceiling" effect, with existing work having demonstrated the subjectivity in rating decisions and identified systematic ratings biases below the ratings implied based on sovereign credit rating methodologies[74,75]. This bias has been partly attributed to geographic and institutional distance between rated EMDEs and the major credit rating agencies, all of which are headquartered in Europe or North America. These issues point to structural biases in their methodology against EMDEs[76], underlining the need for reform of the structure, methodologies and role of credit rating agencies to help scale clean energy investments into EMDEs.

This includes the limits on where this may be suitable or not, included in the following paragraph from lines 476 - 481:

Default rates for private lending in EMDEs are also substantially lower than the default rate implied by historical sovereign credit ratings[78], particularly for infrastructure projects, demonstrating a potential need to decouple the link between project ratings

> *and sovereign credit ratings. Increasing the granularity of information conveyed through sovereign credit ratings from their current single score (e.g., AAA to C-) to incorporate modular assessments of underlying risk factors through a modular sovereign credit rating could support a more risk-informed approach by splitting out the contributions from different risk factors. Modular credit ratings would help ensure that the link between project-level and sovereign credit ratings is more suitably coupled, allowing investors to assess the most applicable country-level factors that could affect the risks of project default (whereas currently all factors and biases within sovereign ratings contribute to the "sovereign ceiling" regardless)[77]. For example, the overall debt burden and debt-to-GDP ratio of the sovereign does not directly affect a privately owned and financed renewables project's viability and risk profile but are an important factor in sovereign credit ratings. Improved project-level data availability will also be essential to informing more risk-reflective behaviour from international investors, alongside scaling domestic capital markets and improving enabling environments for investment. Successful initiatives for the former have included the development of micro-finance institutions and other forms of innovative financing for renewables. For example, local currency loans and the "pay-as-you-go" model provided by Sun King have enabled over 27m solar products to be distributed across Africa (with support from concessional loans from the IFC)[79].*

3) In line 442, page 17 you write "*As corporate and project credit ratings are largely capped at the country's sovereign credit rating (a "sovereign ceiling" for private credit ratings which cannot typically be exceeded73), inaccuracies or misperceptions in sovereign risk can severely constrain project financing conditions. An increasing body of literature has demonstrated the subjectivity and distance bias in sovereign credit ratings for developing countries74,75, as well as structural biases in their methodology against EMDEs76, underlining the need for reform of the structure, methodologies and role of credit rating agencies to help reduce the cost of capital. For example, modular sovereign credit ratings could aid a more risk-informed approach by investors than attempting to reflect a wide range of risk factors into one score (some of which will be applicable to the economic performance of projects or corporates operating under a given sovereign)77*".

Here: a) I am not sure I understand your text. You write that "project credit ratings are largely capped at the country's sovereign credit rating". I understand this as project financed having a base rate defined by the countries sovereign credit rating. Is this correct? Please clarify and improve text for better understanding.

Response: We have reworded the sentences highlighted and expanded them out to a standalone paragraph (lines 460 – 474) to better clarify the links between project and sovereign credit ratings:

Country risk premiums are largely derived from the host country's sovereign credit rating, as investors and businesses typically do not have the ability to conduct their own detailed macroeconomic and political assessments across countries of interest. Sovereign credit ratings act as a hard "sovereign ceiling" where projects or corporations are typically unable to be rated higher than the country rating[73], meaning that they are all but guaranteed a higher cost of capital than the sovereign borrowing costs. Any biases, inaccuracies and/or misperceptions of sovereign risk can therefore severely constrain project financing conditions under the "sovereign ceiling" effect, with existing work having demonstrated the subjectivity in rating decisions and identified systematic ratings biases below the ratings implied based on sovereign credit rating methodologies[74,75]. This bias has been partly attributed to geographic and institutional distance between rated EMDEs and the major credit rating agencies, all of which are headquartered in Europe or North America. These issues point to structural biases in their methodology against EMDEs[76], underlining the need for reform of the structure, methodologies and role of credit rating agencies to help scale clean energy investments into EMDEs.

Further, did I understand if you are suggesting to uncouple the evaluation of individual projects from this sovereign credit rating? The sentence where you talk about distance bias seems to suggest that. Further, it is unclear what you mean by distance bias - please clarify.

Response: We have made our suggestion of uncoupling the evaluation of individual projects clearer, including through the use of modular credit ratings. We have added in the following sentences in a standalone paragraph (lines 476 – 595) to highlight the need to uncouple project- and country-level assessments and discuss the role for modular credit ratings in supporting this:

Default rates for private lending in EMDEs are also substantially lower than the default rate implied by historical sovereign credit ratings[78], particularly for infrastructure projects, demonstrating a potential need to decouple the link between project ratings and sovereign credit ratings. Increasing the granularity of information conveyed through sovereign credit ratings from their current single score (e.g., AAA to C-) to incorporate modular assessments of underlying risk factors through a modular sovereign credit rating could support a more risk-informed approach by splitting out the contributions from different risk factors. Modular credit ratings would help ensure that the link between project-level and sovereign credit ratings is more suitably coupled, allowing investors to assess the most applicable country-level factors that

could affect the risks of project default (whereas currently all factors and biases within sovereign ratings contribute to the "sovereign ceiling" regardless)[77]*. For example, the overall debt burden and debt-to-GDP ratio of the sovereign does not directly affect a privately owned and financed renewables project's viability and risk profile but are an important factor in sovereign credit ratings. Improved project-level data availability will also be essential to informing more risk-reflective behaviour from international investors, alongside scaling domestic capital markets and improving enabling environments for investment. Successful initiatives for the former have included the development of micro-finance institutions and other forms of innovative financing for renewables. For example, local currency loans and the "pay-as-you-go" model provided by Sun King have enabled over 27m solar products to be distributed across Africa (with support from concessional loans from the IFC)*[79]*.*

We have also defined the distance bias by adapting the paragraph on country risk and sovereign credit ratings (lines 460 – 474) to the following, with the relevant passages in bold:

Country risk premiums are largely derived from the host country's sovereign credit rating, as investors and businesses typically do not have the ability to conduct their own detailed macroeconomic and political assessments across countries of interest. Sovereign credit ratings act as a hard "sovereign ceiling" where projects or corporations are typically unable to be rated higher than the country rating[73]*, meaning that they are all but guaranteed a higher cost of capital than the sovereign borrowing costs.* ***Any biases, inaccuracies and/or misperceptions of sovereign risk can therefore severely constrain project financing conditions under the "sovereign ceiling" effect, with existing work having demonstrated the subjectivity in rating decisions and identified systematic ratings biases below the ratings implied based on sovereign credit rating methodologies*****[74,75]*****. This bias has been partly attributed to geographic and institutional distance between rated EMDEs and the major credit rating agencies, all of which are headquartered in Europe or North America. These issues point to structural biases in their methodology against EMDEs*****[76]*****, underlining the need for reform of the structure, methodologies and role of credit rating agencies to help scale clean energy investments into EMDEs.***

Also you write about a modular credit rating (which I assume is the decoupling of overall country risk from individual project risk). Please write in the text a proper definition of this newly introduced term. On that note I have one conceptual comments as well. So what risks would then not be evaluated in the "renewables modular credit rating"? Which risk is relevant for country risk and not relevant for

renewables? I would assume that all country-related risks are important like: currency, rule of law, overall business environment etc.

> **Response:** As discussed above, we have revised this section to include a stronger outline of a modular credit rating in a standalone paragraph talking about the coupling between sovereign and project level (lines 476 – 495). We have also added an example of risk factors at a country level that are not applicable to renewable projects to highlight country-level factors included in sovereign credit ratings that may not be relevant for renewables (lines 479 -491):
>
> *Increasing the granularity of information conveyed through sovereign credit ratings from their current single score (e.g., AAA to C-) to incorporate modular assessments of underlying risk factors through a modular sovereign credit rating could support a more risk-informed approach by splitting out the contributions from different risk factors. Modular credit ratings would help ensure that the link between project-level and sovereign credit ratings is more suitably coupled, allowing investors to assess the most applicable country-level factors that could affect the risks of project default (whereas currently all factors and biases within sovereign ratings contribute to the "sovereign ceiling" regardless)*[77]*. For example, the overall debt burden and debt-to-GDP ratio of the sovereign does not directly affect a privately owned and financed renewables project's viability and risk profile but are an important factor in sovereign credit ratings. Improved project-level data availability will also be essential to informing more risk-reflective behaviour from international investors, alongside scaling domestic capital markets and improving enabling environments for investment.*

4) You write in line 463 about a reform of the overall finance architecture. May I suggest to the authors to stick more to the country level discussion before discussing the global framework. For instance, it would be useful for project developers to have something like a one-stop-shop or preferential treatment for permits and basically local help when getting the project permits, which could help with corruption-related issues.**Response:** We have restructured the discussion so we have the following narrative flow related to risk drivers and interventions in the discussion from line 438 onwards into three paragraphs: 1) Country-level factors, 2) Sovereign credit ratings and the role of modular credit ratings and 3) Interventions from international public finance to include a longer country-level discussion.

> We have included a longer discussion in (1) on country-level factors, including the role of political risk insurance and a one-stop-shop that can help reduce the impact of corruption and other related issues for project developers: We have included a longer discussion in (1 – from line 438 onwards) on country-level factors, including the role of political risk insurance and a one-stop-shop that can help reduce the

impact of corruption and other related issues for project developers. The full paragraph now reads (with the relevant sections to this comment bolded)):

Estimates show that annual clean energy investment into EMDEs will need to increase by more than six times by 2030 for the world to be on track for net zero by 2050[27]. Targeted efforts to bring down the cost of capital in EMDEs will be essential to scaling private investment flows, which our modelling shows are largely held back by high country risk premiums. These conclusions align with the assessment by the IEA that an estimated 70% of the risk factors contributing to high costs of capital in EMDEs are caused by macroeconomic and currency risks[27], accounted for here in the country risk premiums. Factors influencing the country risk premium also include corruption, political structures, high exposure to violence and the strength of legal systems, challenges that pose difficulties in attracting investments beyond just the energy sector. ***Developing a strong enabling environment for investment will be important to scaling private investment to levels required to meet the UN Sustainable Development Goals, including through strengthening domestic institutions, reforming legal systems and designing regulatory environments to promote transparency and fair competition. Existing programmes to support domestic policymakers in addressing these issues include the UNDP's Governance for Anti-Corruption for Peaceful and Inclusive Societies Program and the World Bank's Improving Business Environment for Prosperity Program. For investors, political risk insurance (e.g., through the World Bank's MIGA program) can also provide coverage against country-level risk factors, including expropriation and breaches of contract, whilst for project developers, a dedicated investment facilitation unit for renewable project permitting could substantially reduce developers' exposure to corruption by streamlining approval pathways.***

We have also changed the framing of (3 – lines 489 to 509) to discuss the political and economic challenges to both country-level and the overall finance architecture, which face many challenges and so requires interventions from international finance in the short term:

Domestic measures to improve the enabling environment for investment at a country level and reforms of the global financial architecture to better deliver investment at affordable costs of capital into EMDEs face many economic and geopolitical challenges. In the short term, support from international public financiers (IPFs) will be essential for effectively de-risking clean energy projects and "crowding in" the private investment required, both by serving as an anchor point and addressing underlying policy risk factors[80]. Support can include loan guarantee programmes[81], strengthening national regulatory frameworks[27] and a combination of capacity building and technical assistance[82]. Examples of successful interventions have included risk guarantees and political insurance for Ugandan hydroelectric power[83], support for the development of bankable PPAs in Serbia[84] and efforts to support enabling

environments for private investment into clean energy by the World Bank[85]. IPFs can also provide finance at concessional rates[23], which is critical for the least developed countries that we show here face some of the highest commercial costs of capital. Estimates show that concessional funding for clean energy investment will similarly need to scale up by five to six times to ensure that clean energy investment in developing countries reaches a level that delivers on the Paris Agreement[51,86]. The New Collective Quantified Goal for climate finance, agreed at COP29[87], included an ambition to expand international public and concessional financing but was criticised for lacking sufficient inclusion of grants and other below-market sources of financing[88].

I think the authors have responded in a satisfactory way to the comments made.

We are thankful for your continued engagement with our manuscript draft.
