# Supplementary material for: High costs of capital shape the mitigation effects of renewable energy deployment globally
Source: Nat Commun. 2026 Aug 13;17:9733. doi: 10.1038/s41467-026-75666-6 (PMC13562596; doi:10.1038/s41467-026-75666-6)
Supplement: Supplementary file 3 [file 41467_2026_75666_MOESM3_ESM.docx]

# Extended Data Figures

**ED Figure 1: The annual average capacity factor for renewable energy generation.** Capacity factor is presented in % for a) solar PV and b) wind energy and are generated using the Renewables.Ninja method^62^ and the PV Lib Python module developed by Sandia Labs^61^. The weather data used to generate wind and solar profiles is taken from NASA’s MERRA-2 dataset^99^.

**ED Figure 2:** **Estimates of the nominal weighted average cost of capital (WACC) for 2024, mapped globally, for a) Solar PV, and b) Wind power.** Results are shown on a country-level basis for the 176 countries that had data on country risk premiums available from Damodaran^31^. The WACC was estimated by disaggregating contributions to the cost of debt and equity, advancing on the approach developed by IRENA^55^ (see Methods).

**ED Figure 3: Global technical potential curves for a) solar PV, b) onshore wind and c) offshore wind, ordered by estimated technology and country-specific cost of capital for 2024 and with GDP per capita highlighted.** Note the different scale used for offshore wind electricity potential. Packing densities of 32.95, 6.52 and 4.0 MW per km^2^ were used for solar, onshore wind and offshore wind, with land use constraints applied (see Supplementary). Minimum cut-offs of 10% and 18% average capacity factors across the year were applied to the solar and wind potentials to reflect constraints in economic feasibility^63^.

**ED Figure 4: Levelised cost of electricity from solar PV, plotted globally for a) country-specific costs of capital and b) a uniform nominal cost of capital of 8.2%.** The Uniform cost of capital was taken as the average estimated cost of capital for solar PV in the UNFCCC Annex II countries, which are commonly regarded as the developed country grouping (see Supplementary for the full list of Annex II countries).

**ED Figure 5: Levelised cost of electricity from onshore and offshore wind, plotted globally using a) country-specific costs of capital and b) a uniform nominal cost of capital of 8.1% and 9.0% for onshore and offshore, respectively.** The Uniform costs of capital are taken as the average estimated cost of capital for onshore and offshore wind in the UNFCCC Annex II countries, which are commonly regarded as the developed country grouping (see Supplementary for the full list of Annex II countries).

**ED Figure 6: Average grid electricity carbon intensity, plotted globally for 186 countries and/or regions.** Data is plotted in grams of carbon dioxide per kWh of generated electricity, taken from Ember Climate^48^ for 2023; whilst more recent data for 2024 was available, this was only for 86 countries/regions globally.
